# Being cosmopolitan: evolutionary history and phylogeography of a specialized raptor, the Osprey *Pandion haliaetus*

**DOI:** 10.1186/s12862-015-0535-6

**Published:** 2015-11-17

**Authors:** Flavio Monti, Olivier Duriez, Véronique Arnal, Jean-Marie Dominici, Andrea Sforzi, Leonida Fusani, David Grémillet, Claudine Montgelard

**Affiliations:** CEFE UMR 5175, CNRS - Université de Montpellier - Université Paul-Valéry Montpellier - EPHE, 1919 Route de Mende, 34293 Montpellier cedex 5, France; Department of Life Sciences and Biotechnology, University of Ferrara, via Borsari 46, I-44121 Ferrara, Italy; Réserve Naturelle Scandola, Parc Naturel Règional de Corse, 20245 Galeria, France; Maremma Natural History Museum, Strada Corsini 5, 58100 Grosseto, Italy; Department of Cognitive Biology, University of Vienna, & Konrad Lorenz Institute for Ethology, University of Veterinary Medicine, Vienna, Austria; Percy FitzPatrick Institute, DST-NRF Centre of Excellence, University of Cape Town, Rondebosch, 7701 South Africa; Department of Zoology, University of Johannesburg, P.O. Box 524, Auckland Park, 2006 South Africa

**Keywords:** Accipitriformes, Aves, Cytochrome *b*, Evolutionary Significant Unit, Mitochondrial markers, Molecular dating, ND2, Subspecies

## Abstract

**Background:**

The Osprey (*Pandion haliaetus*) is one of only six bird species with an almost world-wide distribution. We aimed at clarifying its phylogeographic structure and elucidating its taxonomic status (as it is currently separated into four subspecies). We tested six biogeographical scenarios to explain how the species’ distribution and differentiation took place in the past and how such a specialized raptor was able to colonize most of the globe.

**Results:**

Using two mitochondrial genes (cyt *b* and ND2), the Osprey appeared structured into four genetic groups representing quasi non-overlapping geographical regions. The group Indo-Australasia corresponds to the *cristatus* ssp, as well as the group Europe-Africa to the *haliaetus* ssp. In the Americas, we found a single lineage for both *carolinensis* and *ridgwayi* ssp, whereas in north-east Asia (Siberia and Japan), we discovered a fourth new lineage. The four lineages are well differentiated, contrasting with the low genetic variability observed within each clade. Historical demographic reconstructions suggested that three of the four lineages experienced stable trends or slight demographic increases. Molecular dating estimates the initial split between lineages at about 1.16 Ma ago, in the Early Pleistocene.

**Conclusions:**

Our biogeographical inference suggests a pattern of colonization from the American continent towards the Old World. Populations of the Palearctic would represent the last outcomes of this colonization. At a global scale the Osprey complex may be composed of four different Evolutionary Significant Units, which should be treated as specific management units. Our study brought essential genetic clarifications, which have implications for conservation strategies in identifying distinct lineages across which birds should not be artificially moved through exchange/reintroduction schemes.

**Electronic supplementary material:**

The online version of this article (doi:10.1186/s12862-015-0535-6) contains supplementary material, which is available to authorized users.

## Background

The modern distribution of living organisms has been shaped by multiple processes that had profound effects on the dispersal, genetic structure and evolutionary histories of plant and animal populations. Movements of land-masses and successive multiple glacial events that occurred during the Pleistocene caused severe habitat changes which confined many species to warmer refugia and led other taxa to experience demographic reductions or complete extinction [1]. Favourable periods during climatic fluctuations allowed successive population expansions, together with the recolonization of portions of the ancient ranges [2].

Despite the high potential dispersive power of flying birds, it is striking that only a few taxa did colonize most of the world. Excluding seabirds, for which the distribution pattern depends more on ocean basins than on the major land-masses [3], only six landbird species (out of ca. 10,000 species) are known to be cosmopolitan, breeding in each biogeographical region of the world, except Antarctica. This group includes the Great Egret *Ardea alba*, the Cattle Egret *Bubulcus ibis*, the Glossy Ibis *Plegadis falcinellus*, the Barn Owl *Tyto alba*, the Peregrine Falcon *Falco peregrinus* and the Osprey *Pandion haliaetus*.

The Osprey is a medium-sized raptor with flexible breeding habitat requirements across its range. Despite its specialization as a piscivore, it is an opportunistic forager that can feed in both freshwater and marine environments [4]. Northern populations are known to migrate long distances [4, 5], whereas individuals from lower latitudes (e.g. Caribbean, Atlantic islands and Mediterranean basin) are mostly sedentary, or perform small-scale interbreeding movements [4, 6]. One could therefore predict that such broad habitat tolerances and high mobility should homogenize genomes, limiting genetic differentiation across populations at a continental level, as described in other widespread raptors (e.g. *Haliaeetus albicilla:* [7]; *Falco peregrinus:* [8]). However, adult ospreys tend to return to their natal area to breed [9] and such strong philopatry may have played in favour of genetic structuring among populations across the extensive range. Similarly, genetic differences may be expected between long-distance migratory and partially migratory/resident populations.

**Fig. 1:**
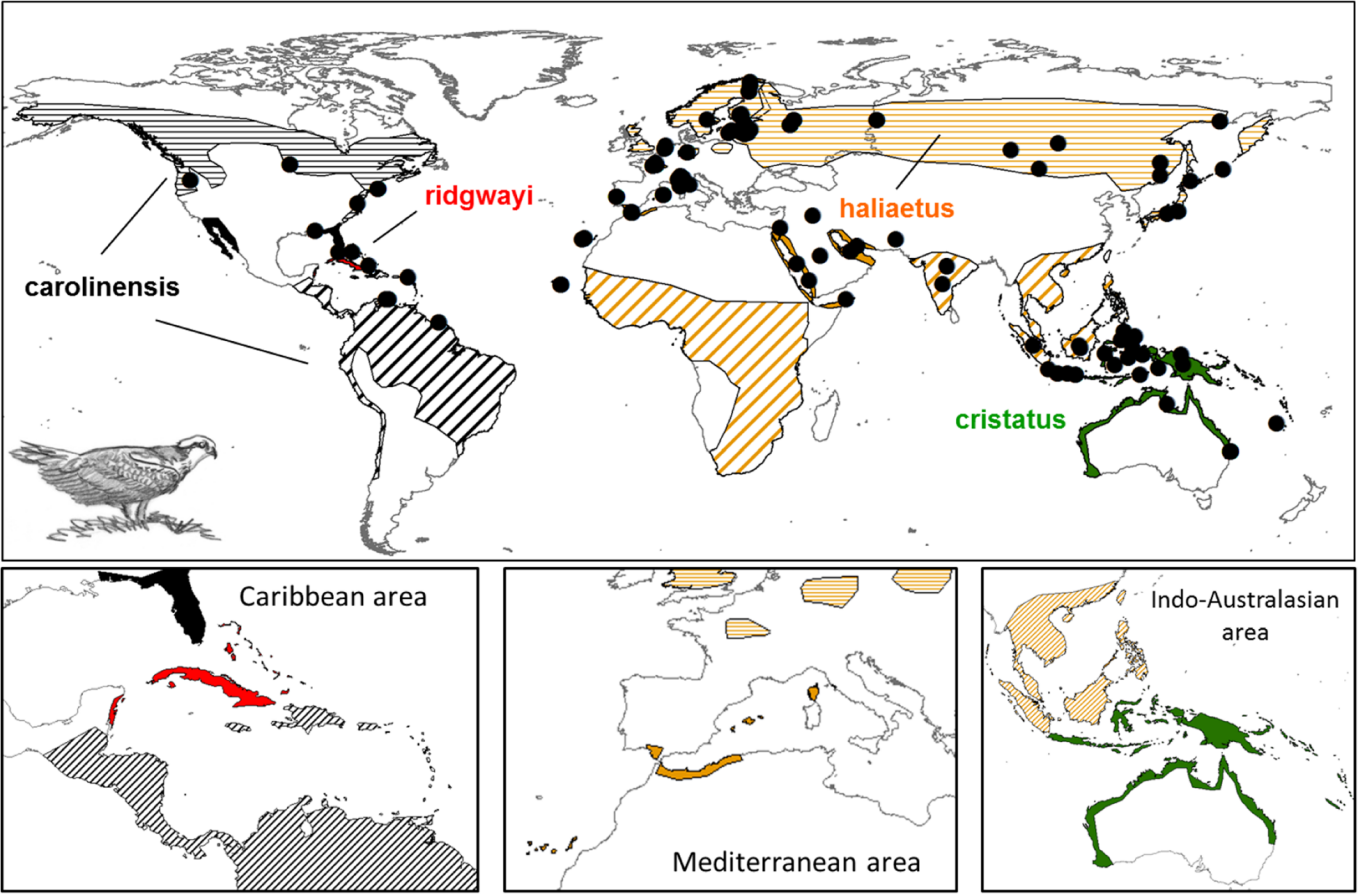
Geographical distribution of *Pandion haliaetus*. Ranges for the four recognized subspecies are in different colors: black for *carolinensis*, red for *ridgwayi*, orange for *haliaetus* and green for *cristatus*. Horizontal stripes are for breeding areas, skew lines for wintering areas and color-filled zones represent areas with sedentary populations. Black circles symbolize sample locations. In the small boxes (from left to right) three zones are zoomed in: Caribbean, Mediterranean and Indo-Australasian areas

On the basis of comparative non-molecular characters such as osteology, pelvic musculature and the distribution of feather tracts, the Osprey is considered sufficiently distinct from other raptor species (family Accipitridae; [10]) to merit a monotypic family (Pandionidae) [11, 12]. The most widely accepted taxonomic arrangement recognises four subspecies of Osprey: *P. h. haliaetus* (Linnaeus, 1758) in the Palearctic from Europe, northwest Africa, and Asia north of the Himalayas, *P. h. carolinensis* (Gmelin, 1788) in North America, *P. h. ridgwayi* (Maynard, 1887) in Caribbean Islands, and *P. h. cristatus* (Vieillot, 1816) in the Indo-Pacific and Oceania (Fig. 1). The four subspecies were traditionally split on the basis of morphometry and plumage characteristics, but the differences are not straightforward [4, 13]. Therefore, referring only to morphology for describing diversity and interrelationships between subspecies has led to controversies in taxonomy.

In this context, using molecular markers is a powerful method for inferring the evolutionary history of the Osprey. Only two genetic studies have been carried out on this species [14, 15], but they did not investigate all subspecies, in the entire distributional range. Here, we carried out an extensive phylogeographic study based on mitochondrial DNA sequences (cytochrome *b* and ND2 genes) from samples covering the worldwide distribution of the Osprey. This exhaustive dataset allowed addressing specific questions. a) Does the Osprey show any phylogeographic structure in mitochondrial DNA at the continental level? b) How was such a specialized raptor able to colonize the entire world? We propose a hypothetical evolutionary scenario explaining how the species’ distribution and differentiation took place in the past. In the light of these new genetic clarifications, we discuss the potential implications for revisions of the taxonomy and for conservation (e.g. defining Evolutionary Significant Units (ESUs; [16]) to design relevant conservation strategies at the appropriate spatial scale.

## Methods

### Sampling, DNA sequencing and alignment

Sampling covered the whole species’ distributional range, with 204 individuals (114 fresh samples + 90 museum specimens) from 31 countries across five continents (Fig. 1; Additional file 1).

Fresh samples (*n* = 114; both blood and feathers) were obtained from wild ospreys at the nest during ringing activities in different breeding sites. For each individual, about 0.5 ml of blood was taken by venepuncture from the wing and stored either on filter blotting papers or in 70 % ethanol in Eppendorf tubes. In some cases, growing body feathers containing traces of blood within the calamus were collected and stored in envelopes.

For areas where it was not possible to collect fresh samples, we completed our sampling using 90 museum specimens. We collected small pieces of skin (about 2 mm from the toepad) from museum study Osprey-skins following the procedure described by Mundy et al. [17]. From each museum, we mainly choose museum study skins of various origins and collected during the breeding season, so excluding possible vagrants, wintering birds or dispersing animals.

From both fresh and museum specimens DNA was extracted and amplified by PCR (see technical procedures below) for the mitochondrial Cytochrome *b* (cyt *b*) and NADH dehydrogenase subunit 2 (ND2). For the cyt *b*, five sequences from the GenBank database were also included, leading to a total of 209 sequences (see Additional file 1). For ND2 a subset of only 37 individuals returned high-quality PCR products to which we added one sequence from GenBank, thus leading to a dataset of 38 individuals (Additional file 1). Finally, four other raptor species were used as outgroups: White-tailed eagle (*Haliaeetus albicilla*), Tawny eagle (*Aquila rapax*), Black-breasted buzzard (*Hamirostra melanosternon*) and Gray-headed kite (*Leptodon cayanensis*). The choice of these outgroups was motivated because 1) both cyt *b* and ND2 sequences were available in GenBank (see [11] for outgroup accession numbers), and 2) they represent four lineages of the Accipitridae family, the sister taxa of Pandionidae (including only *Pandion haliaetus*; [11]). The number of individuals and the length of sequences used for each analysis are reported in supplementary information (Additional file 2).

Total genomic DNA was extracted from fresh and historical samples using a Qiagen DNeasy Tissue kit, following the manufacturer’s instructions (Qiagen, Hilden, Germany). To avoid contamination with contemporary DNA [18], all extractions from museum specimens were performed using the facilities of the platform “degraded DNA” (Labex CeMEB, Montpellier, France) dedicated to degraded DNA experiments, where we adopted the following specific safety measures. Equipment, consumables and Qiagen DNeasy Tissue kits used in the platform were purchased new, while the room was regularly cleaned and exposed to UV overnight after each DNA extraction cycle, in order to destroy possible traces of DNA between successive extractions. Experimentators wore protective clothing and footwear. As a further precaution and following Bantock et al. [18], we worked with a maximum of 12 samples during each series of extraction to reduce the risks of cross-contamination and possible handling errors with tubes. We only used half of each foot-skin sample (about 10 mg of tissue) from which total DNA was extracted. Samples were incubated at least for one night at 56 °C to be digested during the lysis. The tissue was digested in 180 μL buffer ATL/20 μL proteinase K solution for 20-h at 55 °C; other reagents and the spin column were used according to the manufacturer's instructions ("Tissue protocol"), and final DNA elution was performed with 2 × 100 μL of 10 mM Tris, 0.5 mM HC1 pH 9.0 preheated to 70 °C. Multiple negative extraction and amplification controls were carried out simultaneously, using the same instruments and reagents, to detect possible contamination.

Portions of the mitochondrial cyt *b* and ND2 were amplified by PCR. Specific external and internal mitochondrial cyt *b* and ND2 primers were designed in this study for *Pandion haliaetus* (Additional file 3). PCR was performed using 1-μl (fresh samples) to 3-μl (museum specimens) of total DNA extracted, 5-μl of the Qiagen Multiplex PCR kit (containing HotStarTaq DNA polymerase, DNTPs and Multiplex PCR buffer), 1-μl for each primer at 2 pm and 2-μl of purified water. PCR reaction was performed using a MasterCycler Eppendorf thermocycler and began with an initial denaturation of 15 min at 95 °C, followed by 30 cycles of 30 s denaturation at 94 °C, 90 s annealing at 54 °C, 1 min extension at 72 °C and a 30 min final extension at 60 °C. A mitochondrial cytochrome *b* 1040 nucleotides fragment was amplified with PANHF1 and PANHR5 primers; F13 and PHND2-R1 primers were used to amplify a ND2 nucleotide fragment of 1100 bp. In case of degraded DNA, we used internal primers to amplify cyt *b* and ND2 in 300 to 500 nucleotides overlapping fragments (see Additional file 3). Screening of the PCR products was performed by running on a 1 % agarose gel using GelRed TM nucleic Acid gel stain (Biotium). Size products have been compared to long fragments ladder from Eurogentec, Smart LadderTM. After band sizes were determined, PCR products were sequenced at the Genoscope (Evry, France).

Electrophoregrams were read using CodonCode Aligner 4.0.4 software and sequences were aligned by eye using Seaview 4 software [19]. Sequences were also translated into amino acids to check for any stop codons and possible amplification of pseudo-genes. Consensus sequences obtained for cyt *b* and ND2 from both fresh and museum samples were deposited in GenBank under accession numbers given in Additional file 1.

### Partitioning and phylogenetic analyses

Phylogenetic relationships were inferred from the cyt *b* alone or from the concatenated cyt *b* + ND2 datasets. We determined both the best-fit partitioning scheme and the best models of sequence evolution using PartitionFinder 1.0.1 [20].

Phylogenetic trees were reconstructed using two probabilistic methods: Bayesian inference (BI) and maximum likelihood (ML). Bayesian analyses were performed with MrBayes 3.2 [21] using the partitioning strategy obtained with PartitionFinder (see also Results). Two separate runs of five million generations (sampled every 250 generations) were conducted simultaneously. Tracer 1.5 [22] was used to check the convergence between the two runs and to determine the burn-in period. On this basis, the first 2000 phylogenetic trees were discarded (10 %), and the remaining 18000 trees were used to estimate posterior parameters and probability distributions. ML tree was constructed with RaxMl 8.0.17 [23]. As GTR is the only nucleotide substitution model available in RaxMl, GTR + G was applied to all partitions previously determined by PartitionFinder. The robustness of nodes was evaluated with 1000 bootstrap replicates with the option –b. The consensus tree was obtained using the program Consense of the Phylip 3.69 package [24].

Relationships between haplotypes were also visualized as a minimum spanning network, using the Median-Joining (MJ) network algorithm implemented in the program network 4.1.1.0 [25]. In order to avoid artefactual groupings due to missing data, the MJ network was built considering the most complete dataset in terms of nucleotides and individuals, which is a fragment of 661 bp of the cyt *b* on 146 samples.

### Genetic diversity, demographic history and molecular dating

We used Dnasp 5.10 [26] to compute the number of haplotype (nH), haplotype diversity (H), nucleotide diversity (π) as well as the average number of nucleotide differences (k). Mean genetic distances within and between groups were computed using the *p*-distance and a pairwise deletion for the gaps/missing data treatment, as implemented in the Mega 5.10 software [27].

Demographic history of the haplogroups and the whole dataset was determined with different methods. Firstly, R2 [28], Fu’s Fs [29] statistics and their significance were calculated with DnaSP. Ramos-Onsins & Rozas [28] recommended using R2 when population sizes are small (~10) and Fs when sample sizes are large (~50). The historical demography of main haplogroups was also estimated based on the cyt *b* dataset using the skyline plot method (BSP; [30, 31] implemented in Beast 1.8.0. BSP analyses were performed on each group (including all cyt *b* sequences) separately with the cyt *b* partitioned according to codon position and using the HKY + G model as sequence evolution. The likelihood-ratio test performed with tree-puzzle 5.2 [32] rejected the strict molecular clock hypothesis (*p* < 0.05). BSP analyses were thus conducted using a lognormal-relaxed molecular clock with a substitution rate of 0.01973 per lineage per million years as estimated by Nabholz et al. [33] for the Osprey cytochrome *b*. Although this rate has been obtained for the third codon position we apply it because most substitutions (83.3 %) are located at this position in our dataset. Analyses were run for 50 million generations, sampled every 1000 generations, after discarding the first 10 % as burn-in. We used Tracer 1.5 to analyse the results and draw the BSPs.

Time of the most recent common ancestor (TMRCA) was estimated with Beast 1.8.0 based on 23 sequences (19 Osprey haplotypes and 4 outgroups) of the cytochrome *b* (661 positions). The whole alignment was partitioned according to the three codon positions using a HKY + G model of sequence evolution. As previously, analyses were performed using a lognormal-relaxed molecular clock using a substitution rate (ucld. mean) following a normal prior distribution of mean 0.01973 [33] and a standard deviation of 0.005 to take into account some rate uncertainty. Two runs were performed, each of 50 million generations, sampled every 1000 generations, and a 10 % burn-in was applied. The resulting tree files were combined with LogCombiner 1.8.0 and the maximum clade credibility tree (mean height) was obtained with Tree-Annotator 1.8.0.

### Historical biogeography reconstruction

Probabilistic inference of ancestral range was performed using BioGeoBEARS [34] as implemented in R. We performed six different models [34, 35], to reconstruct the biogeographic history of Osprey across continents. Each analysis allows for a different subset of biogeographical possibilities, such as dispersal, vicariance and extinction (see [34, 35]). Accordingly, we tested the following models: Dispersal-Extinction Cladogenesis Model (DEC), Dispersal-Vicariance Analysis (DIVA), Bayesian inference of historical biogeography for discrete areas (BayArea) and the same models including the founder-event speciation parameter (‘j’), DECj, DIVAj and BayAreaj. Finally, statistical fit of the six different models were compared using a model choice procedure by means of the Akaike Information Criterion implemented in the R package BioGeoBEARS [34, 35].

**Fig. 2:**
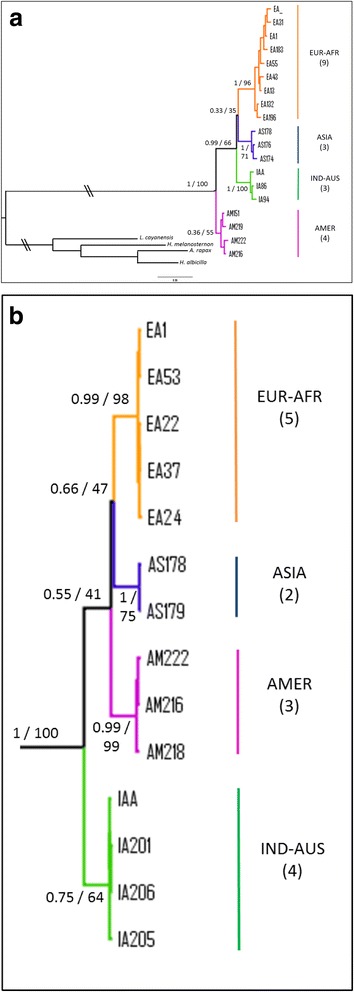
Bayesian phylogenetic trees of ospreys constructed from: **a** 19 haplotypes of cytochrome *b* (661 nucleotides); **b** the concatenated genes (cyt *b* + ND2; 2037 nucleotides, 14 haplotypes). In both trees, four species (Accipitridae family) were used as outgroups. Branch lengths are proportional to the number of substitutions per site and \\ means that branches leading to outgroups have been reduced. For the four main clades the geographic origin of the samples is indicated by different colours and the number of haplotypes is given into parentheses. For supported clades, Bayesian posterior probabilities and ML bootstrap are indicated at nodes, respectively

As input tree, we used the ultrametric tree obtained from the MrBayes (Fig. 2a) analysis on the 19 haplotypes of the cyt *b* gene. We implemented a model including four broad geographic regions: America (A), Indo-Australasia (I), Asia (J) and Europe-Africa (E). The maximal number of areas that could be occupied by one terminal grouping (that is ASIA, AMER, INDIA-AUS and EUR-AFR) was set to two. Analyses were conducted using a non-time-stratified approach under default settings [34]. These six models have been tested in the frame of three different scenarios. The scenario “S0” corresponded to an unconstrained analysis without dispersal matrix. In addition, we tested two alternative biogeographic scenarios by implementing two different geodispersal matrices. In the scenario “S1” we tested a passage from America to Indonesia-Australia (via the Bering Strait and Asian pacific coast) and then towards Asia and Europe-Africa areas in the rest of the Palearctic, whereas in the scenario “S2” the matrix used stipulated a direct dispersal from America to the Old World via the Atlantic Ocean, with a first colonisation of Europe, then extending to Asia and Indonesia-Australia (thus equivalent to a tree in which the AMER and EUR-AFR groups are sister taxa).

## Results

### Phylogenetic relationships

For the mitochondrial cyt *b* dataset (213 individuals = 209 ospreys + 4 outgroups), the best-fit scheme was a partitioning according to codon position with the models HKY + I for position 1 (367 nucleotides), TrN for position 2 (368) and K80 + G for position 3 (368). As the TrN substitution model was not available in MrBayes, the parameter Nst was set to 6. The partitioned ML analysis was performed with 1000 bootstrap replicates using a GTR + G substitution model for each codon position in RaxMl software. The average Bayesian posterior probabilities (pp) and bootstrap values (BP) for supported clades are shown on the tree in Fig. 2. The phylogenetic tree including the totality of individuals (*n* = 209) is reported in supplementary information (Additional file 4).

**Fig. 3:**
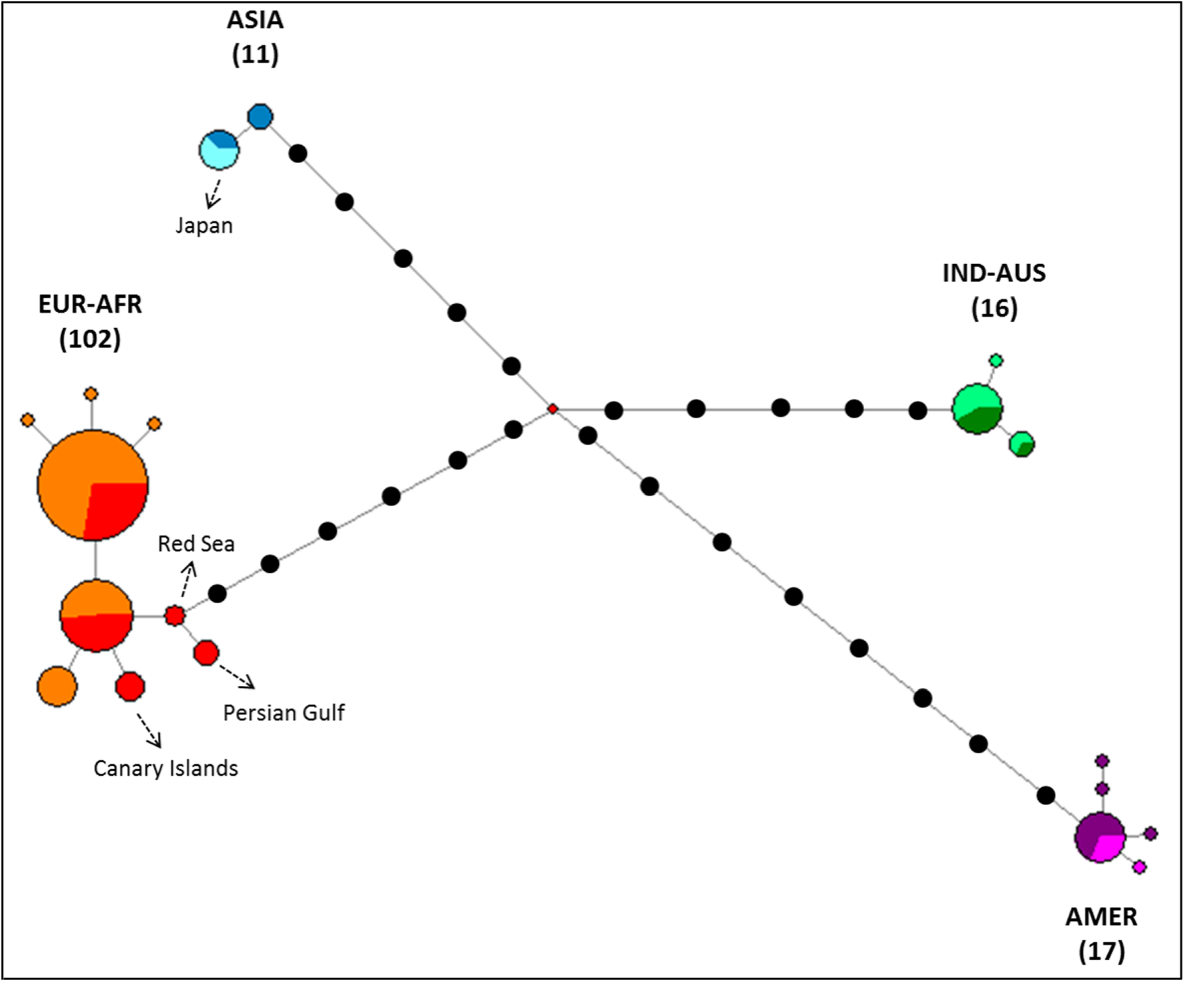
Phylogenetic network of Osprey based on 146 individuals and 661 pb of the cyt *b*. Coloured pies chart indicate different haplotypes with size proportional to the number of individuals. Within each clade, different colours have been used to show the origin of samples: within the EUR-AFR clade, orange is for North and central Europe, whereas red is for Mediterranean, Atlantic Islands and Middle East areas; within the ASIA clade, blue is for Siberia and pale blue for Japan; within the IND-AUS clade, dark and light green are for Indonesia and Australia, respectively; within the AMER clade, violet is for North America and fuchsia for Caribbean. Haplotypes of particular interest (e.g. Japan, Canary Islands, Persian Gulf and Red Sea) are indicated by dotted arrows. Black dots represent missing haplotypes. The median vector is reported with a red dot. The number of individuals is indicated into parentheses for each group

Comparisons on cyt *b* sequences of ospreys (*n* = 146 individuals on 661 bp; see methods) returned 19 mtDNA haplotypes that were used to represent phylogenetic relationships (Fig. 2a). This tree revealed the existence of four groups which represent quasi non-overlapping geographical lineages. A first clade (AMER; pp = 0.36, BP = 55) includes 5 haplotypes from 17 ospreys from the New World: 2 samples from the Pacific coast of USA (Oregon), 8 from the Atlantic coast of USA (Massachusetts and Virginia), 5 from the Caribbean and 2 of unknown origins (one collected in Suriname, South America and the other one from USA without specification). No genetic structure was evidenced and it can be noticed that Caribbean samples (supposed to form a separate group: *ridgwayi* ssp) belong to two haplotypes which are scattered with other *carolinensis* samples (Fig. 3).

The second cluster (IND-AUS; pp = 1.00, BP = 100) is composed of 3 haplotypes including 16 individuals retrieved from the Indo-Australasian area: 6 from various islands of Indonesia and 10 from Australia.

A third group was composed of 2 haplotypes and 11 ospreys from Asia (ASIA; pp = 1.00, BP = 71): 6 from the Pacific coast of Siberia (e.g. regions of Magadan, Khabarovsky, Primorskii and the Kurile islands) and five from Japan (Fig. 3). In addition, two samples that belonged to this haplogroup were collected in other geographic areas: one from continental Asia (Mongolia) and another one from the Indo-Australasian region (New Guinea).

Finally, the largest clade (EUR-AFR; pp = 1, BP = 96) was formed by 102 individuals representing 9 haplotypes, mainly belonging to the Western Palearctic area, with a few exceptions. 93 of these samples were collected in Europe along a latitudinal gradient scattered from northern Europe (Fenno-Scandia and western Russia), central Europe (Germany, France), to southern localities in the Mediterranean area (Corsica, Balearics, Italy, Portugal). Samples from North African coasts (e.g. Morocco) and Atlantic islands (e.g. Canary and Cape Verde) were included in this haplogroup, together with ospreys from the Red Sea and Persian Gulf areas. Three single haplotypes identified particular geographical regions (Fig. 3): Canary Islands (4 individuals), Persian Gulf (2 ind.) and Red Sea (3 ind.). The remaining samples of the EUR-AFR group (93 ind.) mostly belonged to two frequent haplotypes. Interestingly, three geographical exceptions were recorded in this group: two individuals from central Siberia (Tuva and Baikal regions) and one from India.

In total, three potential mixing areas were detected between phylogenetic lineages in the Old world (see Fig. 7): a) one in central Siberia between EUR-AFR and ASIA; b) one in Indonesia between ASIA and IND-AUS and c) a third one between west Indonesia and India between EUR-AFR and IND-AUS.

Despite the different sample sizes, mean genetic *p*-distances within groups (Table 1) were low and showed comparable values (*p* = 0.001 - 0.002), indicating limited genetic variability internal to each lineage. On the other hand, the greatest genetic differences between groups (Table 1) were recorded between AMER and EUR-AFR (*p* = 0.026), whereas lowest values were obtained between IND-AUS and ASIA (*p* = 0.015) and between IND-AUS and EUR-AFR (*p* = 0.017). At the same time, distance between AMER and IND-AUS was smaller (*p* = 0.020) than those between AMER and ASIA (*p* = 0.025).

**Table 1:** Uncorrected pairwise (p)-distance expressed as percentage (%) for cyt *b* within (in bold) and between clades in *Pandion haliaetus*. The number of individuals analzed for each group is given into parentheses

	IND-AUS	EUR-AFR	AMER	ASIA
IND-AUS (37)	**0.1**			
EUR-AFR (131)	1.7	**0.1**		
AMER (26)	2.0	2.6	**0.1**	
ASIA (15)	1.5	2.1	2.5	**0.2**

Relationships between the four haplogroups did not appear well resolved. The structure of the cyt *b* phylogenetic tree (Fig. 2a) revealed that the three Old World lineages (EUR-AFR, ASIA and IND-AUS) formed a rather well supported grouping (pp = 0.99, BP = 66) leaving the AMER group as the basal clade. On the other hand, the sister group relationships between EUR-AFR and ASIA is poorly supported (pp = 0.33, BP = 35). In order to improve resolution between groups we sequenced the ND2 gene for a subsample of 39 individuals (Additional file 4b). The concatenation of the two mitochondrial fragments represented 2037-bp and provided fourteen haplotypes from thirty-eight sequences (Additional file 1). Four partitions were obtained for the best-fit scheme: one for the position 1 of cyt *b* (with the model K80 + G), one for the cyt *b-*position 2 and ND2-position 3 (model HKY + I), one for the cyt *b-*position 3 and the ND2-position1 (model TrN) and one for the ND2-position 2 (model K81uf + G). As previously stated, the TrN model was approximated with Nst = 6 in MrBayes. The analysis carried out on the two genes (Fig. 2b for the 14 haplotypes and Additional file 4c for the 38 individuals) reinforced the support for the four main lineages, although it should be noted that the ASIA group was now represented only by five samples from Japan. Even though the number of nucleotides has been doubled, the only noticeable gain is a slight increased support for the node EUR-AFR/ASIA (pp = 0.66, BP = 47) whereas other relationships, including the position of the root, remained unresolved.

### Network, genetic variability and demography

The cyt *b* network (Fig. 3) confirmed that four major groups which were included in 19 unique mtDNA haplotypes. The EUR-AFR clade (*n* = 102) resulted in nine haplotypes differing by only one nucleotide change. Two out of the nine haplotypes were most frequent, and shared by the majority of the individuals (58 and 25 individuals, respectively). Despite remarkable differences in breeding and movement ecology, Osprey populations of lower latitudes within the EUR-AFR did not show notable haplotypic differences when compared to the northern and central European birds. The four samples from the Canary Islands shared a single haplotype (Fig. 3). Within the IND-AUS group (*n* = 16) only three haplotypes were found, differing by only one nucleotide position. Five haplotypes were observed within the AMER group and 13 samples out of 17 showed the same haplotype, which was shared by ospreys from both western and eastern USA and from the Caribbean. Finally, within the ASIA group (*n* = 11) two haplotypes were recorded. The five samples from Japan were characterized by a single haplotype, which was shared with 2 samples from East Siberia.

Despite slight variation within each group (haplotypes were mainly distant by only one or two positions), a larger number of nucleotide differences were recorded between clades. The AMER group recorded the greatest genetic distance with EUR-AFR (a minimum of 15 nucleotides changed), whereas the minimum number of changes is 11 positions between IND-AUS and ASIA (Fig. 3).

**Fig. 4:**
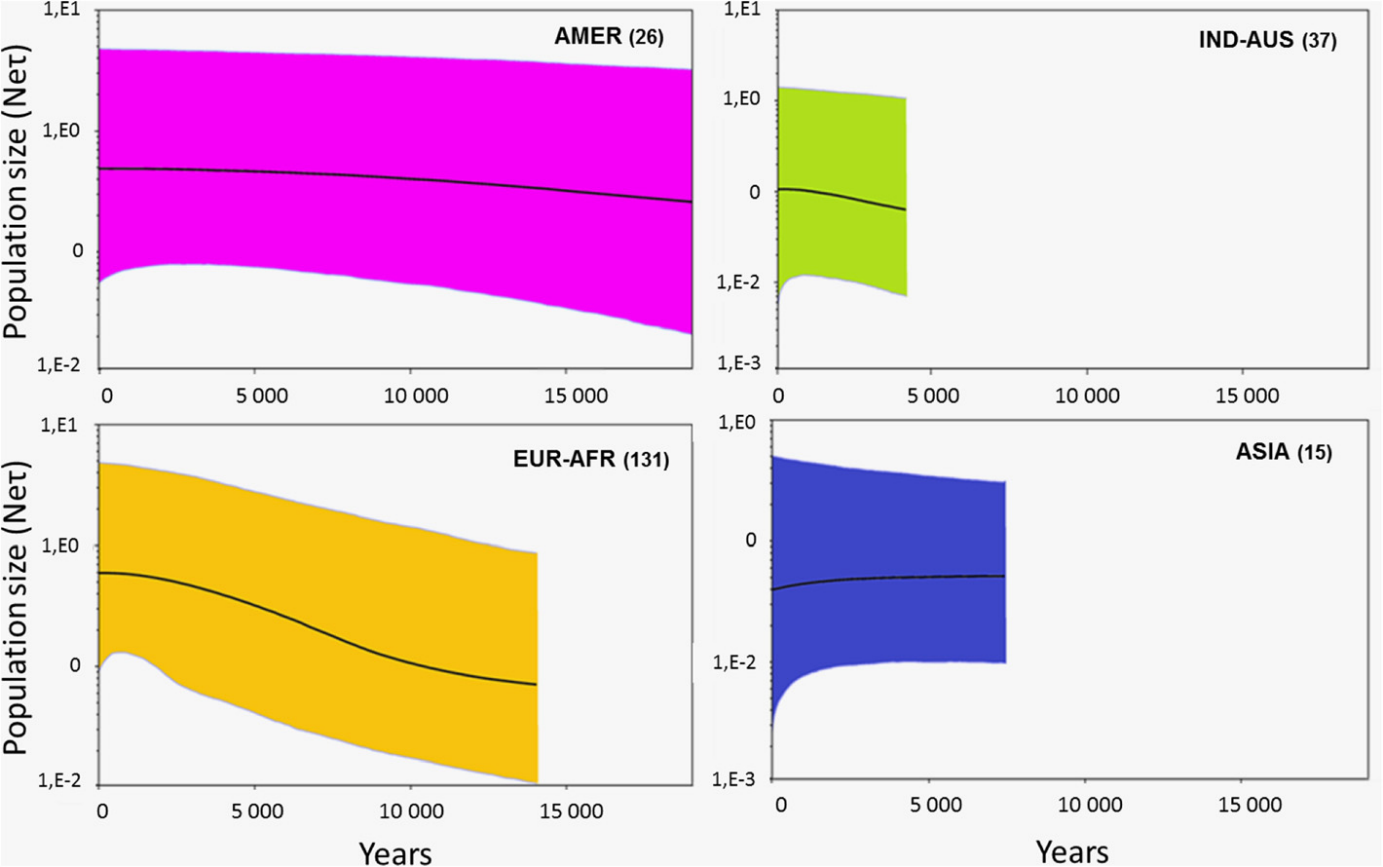
Bayesian skyline plots showing the demographic histories of the four main haplogroups identified in the *Pandion haliaetus* sequences. On the x-axis the (inversed) time is expressed in years. The population size (expressed in units of Neτ, the product of the effective population size per generation length) is reported on the y-axis. The number of individuals analyzed for each group is given into parentheses

In the subset of 146 ospreys, 34 polymorphic segregating sites were discovered within the 661 bp cyt *b* fragment. The haplotype diversity (H), nucleotide diversity (π) and other statistics were computed for the four recognized haplogroups and the whole dataset combined (Table 2). Haplotype diversity was higher in the largest group of EUR-AFR (H = 0.615) and lower for the three other groups (range: 0.425-0.436). The nucleotide diversity showed similar patterns between groups, being very weak in each lineage (range: 0.00066-0.00138). Overall, H was 0.795 and π was 0.0106 for all *Pandion* samples. Demographic history of the four phylogroups, as inferred on the basis of Fu’s F_S_ and R2 statistics (Table 2), indicate that only the AMER group yielded very significant values for both indices, whereas the EUR-AFR lineage showed a significant value for the Fu’s FS only. Thus, population expansion can be assumed for the American and possibly for the Western Palearctic groups. By comparison, Bayesian skyline plots (Fig. 4) indicated that the two haplogroups AMER and IND-AUS remained demographically stable whereas the EUR-AFR haplogroup showed a trend of demographic expansion starting >10,000 years ago. Conversely, the ASIA haplogroup was the only one showing a pattern of demographic decline although demographic stability cannot likely be ruled out (Fig. 4).

**Table 2:** Estimates of across and within-population variability of cyt *b* sequences of Osprey mtDNA

*Phylogroups*	n	n_*H*_	H (s.d.)	π (s.d.)	k	*Fs*	R_2_
Overall	146	19	0.795 (0.026)	0.01064 (0.00087)	6.872	1.413	0.100
AMER	17	5	0.426 (0.147)	0.00087 (0.00035)	0.574	−2.826***	0.099***
EUR-AFR	102	9	0.615 (0.043)	0.00138 (0.00015)	0.901	−3.44*	0.056
ASIA	11	2	0.436 (0.133)	0.00066 (0.00020)	0.436	0.779	0.218
IND-AUS	16	3	0.425 (0.133)	0.00068 (0.00023)	0.450	−0.571	0.145

Sample size (n), number of haplotypes (nH), haplotype diversity (H), nucleotide diversity (π), and the average number of pairwise differences (k). The value of the Fu’s Fs test and R2 of Ramos-Onsins & Rozas [28] are also reported; stars indicate significant values (*p < 0.05 and ***p < 0.001)

### Molecular dating and biogeographical inference

**Fig. 5:**
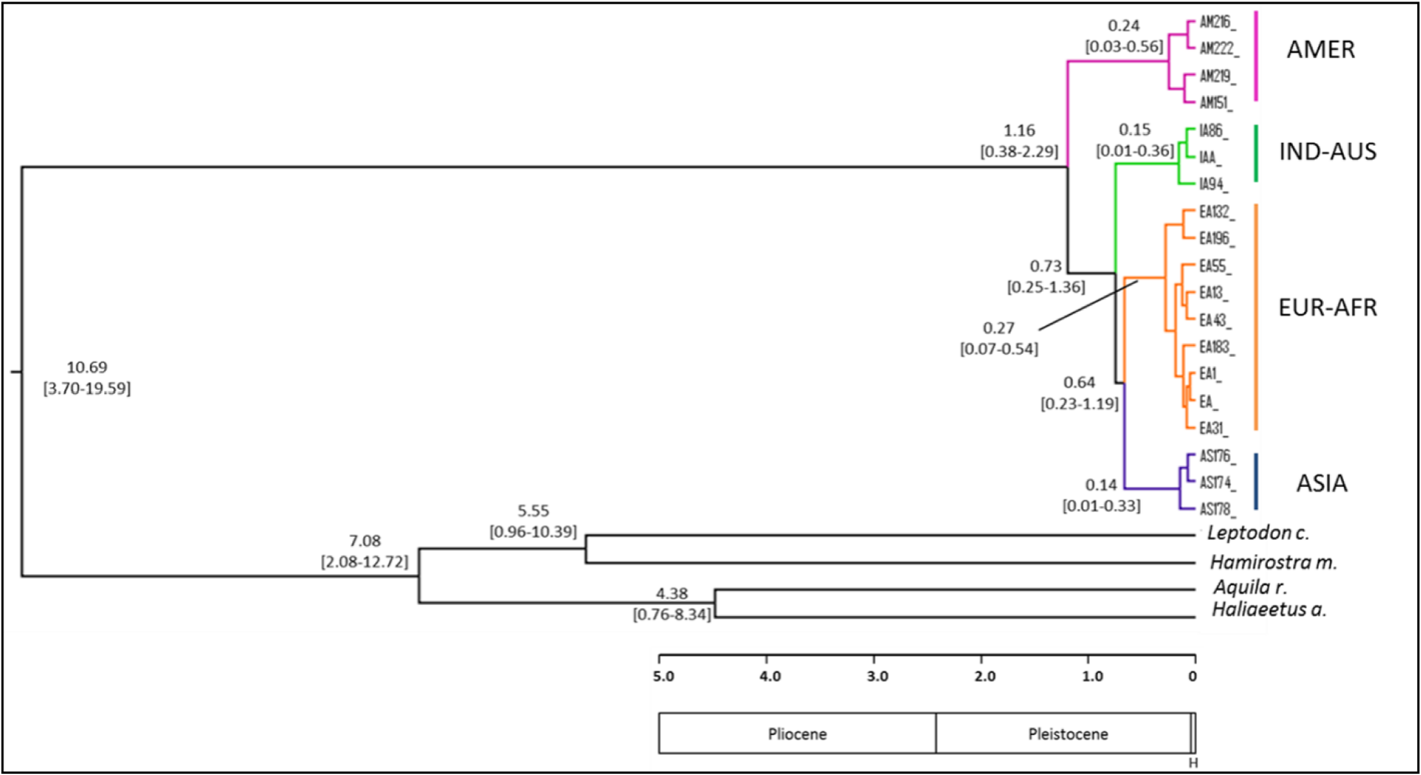
Chronogram obtained with BEAST 1.8.0 showing the time of divergence for the diverse splits in *Pandion haliaetus* using a substitution rate of 0.01973 ± 0.005 per lineage per million years. Values between brackets at nodes indicate 95 % highest posterior densities. The colour code used for each haplogroup is the same as in previous figures. A partial geological time scale is reported below the chronogram (H = Holocene)

Diversification for each of the four groups was dated between 0.14 and 0.27 Ma, that is during the Upper Pleistocene. Among the four lineages, the initial split that individualized the AMER group occurred about 1.16 Ma in the Early Pleistocene (Fig. 5). Subsequent events concerned firstly the divergence of the IND-AUS group at 0.73 Ma whereas the last divergence event that generated the ASIA and EUR-AFR clades occurred at about 0.64 Ma. These two last events can be considered as nearly concomitant considering the large overlapping of the divergence dates (see 95 % HPD on Fig. 5).

**Fig. 6:**
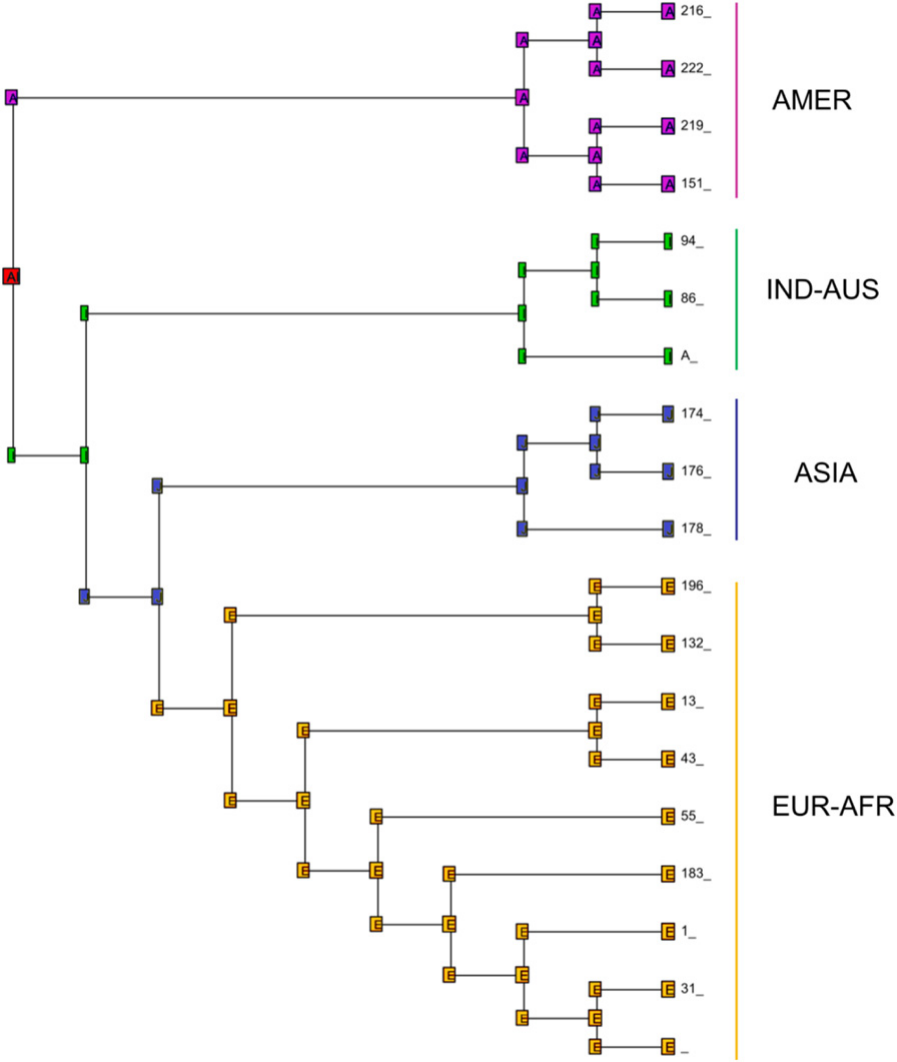
Ancestral-area estimations (above nodes) for *Pandion* evolution, using the unconstrained DIVA + J model in BioGeoBEARS, according to the selected scenario “S1”. Letters represent the areas used for the biogeographical reconstructions: A = America (violet), I = Indo-Australasia (green), J = Asia (blue) and E = Europe-Africa (orange); in red the common ancestor between A and I. Outgroup species used to root the tree are not shown

Among the six models tested in biogeographic inference, the ancestral area reconstruction method using the likelihood dispersal-vicariance analysis with the founder parameter (DIVAj) had a higher probability of being the best model for each of the three scenarios tested (Table 3). With this model, the proposed scenario “S1” returned the best likelihood values. The “j” parameter for founder-event significantly increased the likelihood of the model (DIVA lnL = − 10.3, DIVA + j lnL = − 7.3, *P* = 0.013) because “j” was estimated to be greater than zero (j = 0.108) whereas the estimated rate of both dispersal and extinction was essentially zero (d = 0; e = 0; Fig. 6; Table 4). The ancestral reconstruction (Fig. 6) thus favoured the scenario in which the radiation of *Pandion* started from America and expanded towards the Old World with subsequent founder events (j); the ancestor (AI) first diversified in the Indo-Australasian area by vicariance and then underwent a range expansion to occupy the Eastern Asia (J) and Western Palearctic (E).

**Table 3:** Comparison of the six biogeographical reconstruction models for three different scenarios (“S0”, “S1”, “S2”; see text for definition)

	*Scenario S0*			*Scenario S1*			*Scenario S2*		
Model	lnL	AIC	ῳ_i_	lnL	AIC	ῳ_i_	lnL	AIC	ῳ_i_
DEC	−13.984414	31.97	0.042	−13.072694	30.15	0.018	−14.54651	33.09	0.55
DEC + J	−9.861326	25.72	0.96	−8.059002	22.12	0.98	−13.75296	33.51	0.45
DIVALIKE	−11.852773	27.71	0.11	−10.371666	24.74	0.11	−13.23454	30.47	0.66
**DIVALIKE + J**	**−8.73571**	**23.47**	**0.89**	**−7.315617**	**20.63**	**0.89**	**−12.90696**	**31.81**	**0.34**
BAYAREALIKE	−17.770147	39.54	0.0047	−18.357268	40.71	0.0002	−19.58099	43.16	0.025
BAYAREALIKE + J	−11.405964	28.81	1	−8.920927	23.84	1	−14.90445	35.81	0.98

For each model of each scenario are indicated the log-likelihood (lnL), the Akaike information criterion (AIC) values, and the Akaike weight ῳ_i_ (indicating the relative likelihood of the model). The model with lowest AIC value is marked in bold font and the most likely scenario is underlined. DEC = Dispersal-Extinction Cladogenesis; DIVA = Dispersal-Vicariance Analysis; BAYAREALIKE = Bayesian inference of historical biogeography for discrete areas; j = founder-event speciation parameter

**Table 4:** Details of parameter estimations (dispersal (*d*), extinction (*e*), and founder-event speciation (*j*)) from the analyses of biogeographic inference of scenario S1 in BioGeoBEARS.The model with lowest AIC value is marked in bold font

Model	lnL	Number of Parameters	AIC	*d*	*e*	*j*
DEC	−13.072694	2	30.145388	4.67e-02	1.59e-02	0.000000
DEC + J	−8.059002	3	22.118004	1.00e-12	1.00e-12	0.126265
DIVA	−10.371666	2	24.743332	4.30e-02	1.00e-12	0.000000
**DIVA + J**	**−7.315617**	**3**	**20.631234**	**1.00e-12**	**1.00e-12**	**0.1079407**
BayArea	−18.357268	2	40.714536	5.90e-02	7.31e-02	0.000000
BayArea + J	−8.920927	3	23.841854	1.00e-07	1.00e-07	0.1458791

The model with lowest AIC value is marked in bold font.

## Discussion

### Species diversity and demography

Our study revealed that the Osprey is structured into four main genetic groups, representing quasi non-overlapping geographical regions. Each lineage, though including birds from distant geographic areas, showed low internal genetic variability as revealed by the very low values (range: 0.1-0.2 %; Table 1) observed for the mean intra-group genetic distances. Haplotypic and nucleotide diversities were also weak, with only 9 haplotypes observed for the largest group including 102 ospreys (EUR-AFR), and values were even smaller for the other three lineages (Table 2). The two most common haplotypes in the clade EUR-AFR showed high overlap in ranges (e.g. individuals from very distant populations, like Finnish and Moroccan birds, were included in the same haplotype). A marked geographic substructuring was recorded only for individuals of the Canary Islands, Red Sea and Persian Gulf areas, each one representing a single haplotype (even if sample size is low; Fig. 3).

Despite the low variation within each group, the network (Fig. 3) revealed a high number of nucleotide differences between the four distinct clades. Overall, the mean sequence divergence across all populations (the entire *Pandion* mix) is 1.2 %, which is higher than the values recorded for the Red kite *Milvus milvus* (0.75 % observed for the mitochondrial DNA control region; [36] or the white-tailed eagle *Haliaeetus albicilla* (0.7 % for mitochondrial DNA control region; [7]). The mean genetic distance between groups (range: 1.5-2.6 %) is comparable to, or even greater than, those observed for the mitochondrial cyt *b* in several members of closely related sister eagle species from the genera *Aquila*, *Hieraaetus* (range: 1.7-2.1 %; [10]) and *Haliaeetus* (range: 0.3-9.8 %; [37]).

Populations within each group were poorly differentiated, suggesting that they might have experienced a reduction of genetic variation. Such low values are usually related to populations that experienced demographic crashes or remained isolated in fragmented habitats [38, 39]. However, the historical demographic reconstruction for each lineage suggested that they did not experience any strong bottleneck phases (Fig. 4), but rather underwent stable trends or slight increases.

For the EUR-AFR clade, Bayesian skyline curves (Fig. 4) suggest that populations encountered a recent expansion, which probably started about 10,000 years ago. This fits well with a recent review of the Holocene fossils of Osprey in central Europe from 10,000 years BP [40]. In Europe, the Osprey expanded its distribution area in the following centuries until the beginning of the 20^th^ century. Then, despite declines during the 1960-70s, populations were able to recover, showing positive demographic trends in most areas [41]. On the other hand, the ASIA clade seems to be the only one that potentially suffered some demographic decline (Fig. 4). The same trend is also suggested by very low nucleotide diversity and a positive Fs value. However, such values might also be related to the small sample size of this group (15 samples). This result needs hence to be confirmed by further analyses with more extensive sampling from East Asia.

Why then does each clade show such low genetic variability? Low levels of genetic variation can be the consequence of recent population declines, or represent an ancestral condition, inherited from an ancient evolutionary history [42]. To bring support to these two hypotheses, we compared haplotype diversity between museum and fresh specimens among the EUR-AFR group (*n* = 102). We found that for 10 oldest museum specimens (dated between 1872 and 1959; see Additional file 1), most haplotypes (7 out of 9) are also represented in the fresh samples, thus suggesting that there has been no recent loss of haplotypic diversity. Several studies have also reported stable genetic diversity despite declines in population size (e.g. [43]). For example, no obvious loss of genetic diversity was detected among Canadian peregrine falcons (*Falco peregrinus*) despite a population bottleneck in the 20^th^ century [44]. In our case, one possible explanation is the colonization of new areas by few individuals carrying only a few mitochondrial lineages from the genetic pool (founder effect). Furthermore, source populations could have experienced a reduction in genetic variability due to climate fluctuations during the Quaternary; remnant populations in refugia represented the genetic source for the following recolonization (see below).

### Phylogenetic inferences

The cyt *b* phylogenetic tree (Fig. 2a and Additional file 4a) suggests that the AMER haplogroup constitutes the first genetic group that emerged among the four groups evidenced for *Pandion haliaetus*. This relationship is more supported by the cyt *b* alone (1103 bp) than by the ND2 alone (1078 bp; Additional file 4b). Moreover, the combination of cyt *b* and ND2 (2037 bp, Fig. 2b) did not increase markedly the resolution, thus suggesting that the divergence of the four major clades occurred over a relatively short time period. Another explanation could be that irresolution arose as a misplacement of the root of the tree due to the use of too divergent outgroups. Indeed, there are no optimal outgroups available for the phylogeny rooting of *Pandion*, since it constitutes a long branch in the phylogeny of raptors, distant from its sister Accipitridae family [11, 45]. In preliminary analyses we also included other outgroups such as *Sagittarius serpentarius* and/or different species from the Cathartidae family, but no evident improvements in branching were recorded. Similar problems of rooting have been reported in other phylogeography studies [46, 47].

**Fig. 7:**
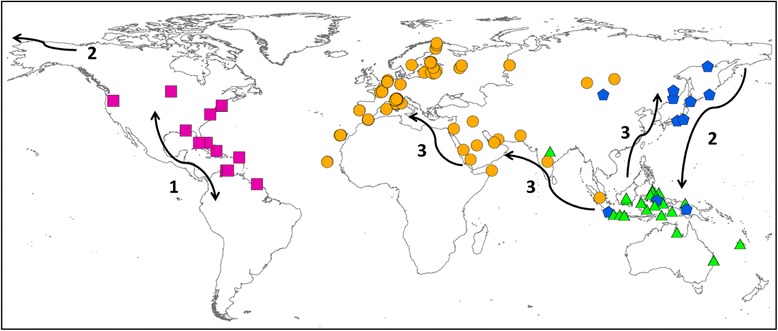
Geographical distribution of the four haplogroups of *Pandion haliaetus*. Symbols and colours indicate both sample locations and genetic group: violet squares for AMER, orange circles for EUR-AFR, blue stars for ASIA and green triangles for IND-AUS (see results). Numbers with their corresponding arrows describe the different phases of the hypothetical colonization scenario

Nevertheless, other arguments can be advocated to reinforce the hypothesis that the Osprey originated in the New World (step 1 in Fig. 7). First, the oldest recognized Osprey specimen is a *Pandion homalopteron* of the mid-Miocene of California dated at 13 Ma [4]. This is in accordance with our molecular dating which estimated at ca. 10.69 Ma (95 % HPD: 19.59-3.70) the origin of a first ancestor for *Pandion sp* (Fig. 5). In addition, as far as we know no fossil was found in Australia (where the species is frequent today), whereas seven fossils from late Pleistocene have been found in Florida [40]. Second, calculation of the *p*-distances between groups (Table 1) indicates that the AMER group is the most divergent compared to the other three groups, suggesting its more ancient origin (and a closer relationship with IND-AUS group). Our molecular dating estimated at 1.16 Ma (Pleistocene) the diversification of *Pandion haliaetus*. Further, the phylogenetic trees (see also scenario “S2” in Table 3) did not support a sister group relationship between AMER and EUR-AFR, as it would be expected in the case of a direct colonization from America to Europe (across the Atlantic Ocean). On the contrary, a pronounced phylogenetically old separation between American and Western Palearctic populations emerged, in accordance with previous studies [10, 15].

### Biogeographic inferences

The major result yielded by phylogeographic inferences is a split of *Pandion* in four clades that appear to have diverged from each other within relatively short time. Because of the uncertainty with regard to the relative branching order of these clades (see Results), patterns of colonization phases should be considered as tentative.

The best biogeographical scenario supports America as the most likely area of origin of *Pandion* (step 1 Fig. 7). The selected model (DIVAj) favours an expansion of Osprey populations that underwent diversification by subsequent founder events followed by vicariance processes. Synthetically, from America, Osprey should have colonized the Indo-Australasian region passing through the Pacific coast of Asia (step 2 Fig. 7). From refugia located in Indonesia-Oceania, rapid range expansion allowed the settlement in eastern Asia and the Western Palearctic (Fig. 5, Fig. 6, step 3 Fig. 7). As a supporting case, cyt *b* data suggest that Japan was likely colonized after Russia and not directly from Australia. Such a scenario would explain the current distribution of *Pandion* across all continents and the genetic divergence found among the four lineages. This hypothesis is corroborated by the *p*-distances which displayed lower values between AMER and IND-AUS (*p* = 0.020) than those recorded between AMER and EUR-AFR (0.026). This result indicates that the EUR-AFR group did not originate directly from the AMER group, as also suggested by the lowest likelihood values yielded by the alternative biogeographical reconstruction (scenario “S2” in which EUR-AFR would have been colonized from AMER; Table 3).

### Implications for taxonomy and conservation

The four genetic groups that we found do not entirely correspond to the four subspecies described, based on morphological characters [4, 13]. The IND-AUS lineage fully matched geographically with the subspecies *cristatus* [48]. Contrary to current taxonomy, North American *carolinensis* ospreys did not differ from Caribbean *ridgwayi* birds as there is no evident structure in the phylogeographic tree and in the network. On the other hand, in Eurasia, we discovered that the subspecies *haliaetus* was actually composed of 2 lineages (EUR-AFR and ASIA) that are undistinguishable morphologically. This reflects the poor knowledge of the species in Asia where detailed information about biology and distribution are needed [49]. More samples should be collected to clarify the geographical limits of these lineages, especially in the regions where we found a zone of overlap (e.g. central Siberia with EUR-AFR clade and in Indonesia with IND-AUS clade).

Overall, genetic distances between Osprey clades (Table 1) are in a range which has already been used by taxonomists for designating distinct raptor species (e.g. [37]). However, we detected a relatively low overall nucleotide diversity (1.0 %) compared to other large raptor species with a similar wide distribution (e.g. *Gypaetus barbatus*, 2.9 % for the control region; [46]; but see [7] for *Haliaetus albicilla*, 0.7 %). It might be objected that more variability would potentially have been found by including more geographic samples for the different clades. However, if we consider the EUR-AFR clade (131 individuals covering a large geographic area), the variability was as weak as for other groups that are represented by a much smaller number of birds (15 to 37). The decision for splitting ospreys into different species (e.g. the split of *P. h. cristatus* as a full species called Eastern Osprey; [50]) should integrate also other factors besides morphology and mtDNA differences; e.g. nuclear genes and behavioural aspects, including migrations that could play an important role as reproductive barriers between distant populations [50].

A first step towards a sound global management and conservation plan is to define Management Units (MUs) and Evolutionary Significant Units (ESUs; [16]). As a matter of fact, subspecies have often been used as proxies for units of conservation in absence of a genetic data indicating distinct evolutionary units [51]. Our results evidenced four different lineages that may deserve the status of ESUs for specific management actions. A better knowledge of the distribution range of each lineage is strongly needed in the near future. In particular, the ASIA lineage should be a priority target for multiple reasons: 1) this lineage has never been described before; 2) it relied on a limited number of samples (*n* = 15) from only a few areas; 3) the majority of the samples analysed were museums specimens, so the current presence of this lineage in East Russia and Indonesia needs to be confirmed; 4) it is the only one clade showing signals of demographic decline; and 5) no clear morphological characteristics can presently help identification.

## Conclusions

Our study revealed that, at the global scale, the particular evolutionary history of the Osprey has partitioned the species into 4 distinct clades with clear genetic differentiation. In addition, our findings indicate that broad habitat requirements and high mobility of Osprey were more important factors than philopatry in shaping genetic diversity at the intra-clade level. Further genetic study using microsatellite markers are ongoing (Monti et al., in prep.) in order to reveal more recent differences and to quantify gene flow among populations that show differences in migratory or reproductive behaviours (e.g. North and Central European populations vs Mediterranean, Canary Islands, Cape Verde and Red Sea).

Even though the Osprey is currently globally listed as “Least Concern” according to IUCN criterions [52], it is considered a priority species for conservation across its distributional range. Indeed, the Osprey is taxonomically unique and conserving its phylogenetic diversity should be a priority [53–55]. The species experienced a severe decline during the 19th and 20th century that led to significant demographic declines and local extinctions [56–58]. Consequently, the Osprey has emerged as an important flagship species and during recent decades has been involved in 25 reintroduction projects in the USA [9, 58] and in Europe [57, 59]. Our results suggest that future reintroduction projects should be conducted using source populations from the same lineage (e.g. within Europe birds chosen for translocation should originate from the Western Palearctic, avoiding individuals belonging to the other lineages). However, before concluding that no restriction needs to be adopted for translocations between populations within the Western Palearctic, other ecological, demographical or behavioural variables should be considered.

### Ethics statement

A specific ethical committee was not required for this study. Bird handling was performed under animal experimentation permits 34–369 (David Grémillet) delivered by the French ‘Direction Départementale de la Protection des Populations’ and under the licence of Olivier Duriez from the Centre for Bird Population Studies (CRBPO) of the Natural History Museum (MNHN, Paris): according to the French law of 22 September 2008, the CRBPO has the delegation by the Ministry of Ecology, Energy, Sustainable Development and Land Settlement for allowing the owners of a general bird ringing licence to capture and handle birds from protected species, and collect samples or mark them (with rings or any other device like GPS units).

### Availability of supporting data

The data set supporting the results of this article is included within the article and its additional files “Additional files 1, 2, 3 and 4.docx”. Nucleotide sequences have been submitted to GenBank and accession numbers are provided in Additional file 1. Alignments used for each analysis (please refer to the table in Additional file 2 for matching) have been included as Additional file 5.

## Additional files

Additional file 1:
**Taxon sampling.** Detailed list of the 209 Osprey samples indicating: sample lab code, subspecies according to morphology classification, country of collection, locality, sample type (tp = toepad; wb = wet blood; db = dry blood; ft = feather; fs = fasta sequence), codes, gene bank number accession for cyt *b* and ND2) and name of the institution and/or collector (with affiliation). (DOC 370 kb) Additional file 2:
**Sampling for analyses.** Number of individuals (N) and sequence length (L in base pairs) used for each analysis (phylogeny and network) and for each gene or concatenation of genes. nd means not done. (DOC 29 kb) Additional file 3:
**DNA amplification.** Cytochrome *b* and ND2 primer names and sequences for amplification and sequencing. (DOC 32 kb) Additional file 4:
**Phylogenetic trees of cyt** ***b*** **, ND2 and cyt** ***b*** **+ ND2.** Bayesian phylogenetic trees of ospreys constructed from: a) the cytochrome *b* (209 sequences, 1103 nucleotides) showing the four supported clades as well as the geographic origin of the samples; b) the ND2 (39 sequences, 1078 nucleotides) and (c) the concatenated genes (cyt *b* + ND2; 38 sequences, 2037 nucleotides). In all the trees, four species belonging to the Accipitridae family were used as outgroups. Branch lengths are proportional to the number of substitutions per site and \\ means that branches leading to outgroups have been reduced. For supported clades, bayesian posterior probabilities and ML bootstrap are indicated at nodes, respectively. (DOC 182 kb) Additional file 5:
**Alignments used for each analysis:** SI1 = cyt *b* (209 sequences, 1103 nucleotides); SI2 = ND2 (39 sequences, 1078 nucleotides); SI3 = cyt *b*+ND2 (38 sequences, 2037 nucleotides); SI4 = cyt *b* (19 haplotypes, 661 nucleotides); SI5 = cyt *b*+ND2 (14 haplotypes, 2037 nucleotides); SI6 = cyt *b* network (146 sequences, 661 nucleotides). Refer to the table in Additional file number 2 for matching. (ZIP 27 kb)
