# Supplementary material for: Being cosmopolitan: evolutionary history and phylogeography of a specialized raptor, the Osprey Pandion haliaetus
Source: BMC Evol Biol. 2015 Nov 17;15:255. doi: 10.1186/s12862-015-0535-6 (PMC4650845; doi:10.1186/s12862-015-0535-6)
Supplement: Additional file 1: — Taxon sampling. Detailed list of the 209 Osprey samples indicating: sample lab code, subspecies according to morphology classification, country of collection, locality, sample type (tp = toepad; wb = wet blood; db = dry blood; ft = feather; fs = fasta sequence), codes, gene bank number accession for cyt b and ND2) and name of the institution and/or collector (with affiliation). (DOC 370 kb) [file 12862_2015_535_MOESM1_ESM.doc]

**Additional file 1: Taxon Sampling**

Detailed list of the 209 Osprey samples indicating: sample lab code, subspecies according to morphology classification, country of collection, locality, sample type (tp = toepad; wb = wet blood; db = dry blood; ft = feather; fs = fasta sequence), codes, Genbank number accession for cyt *b* and ND2) and name of the institution and/or collector (with affiliation).

| **Sample Code** | **Subspecies** | **Country of Collection** | **Locality** | **Sample Type** | **Collection date** | **Ring/Museum Code** | **Genebank A.N. cyt *b*** | **Genebank A.N. nd2** | **Institution and Reference** |
| --- | --- | --- | --- | --- | --- | --- | --- | --- | --- |
| 70 | ridgwayi | Netherland Antilles | Netherlands Antilles-Curacao | museum/tp | 01/11/1953 | ZMA.AVES.28711 | KT123852 |  | Naturalis Biodiversity Centre - Leiden, NL (Becky Desjardins) |
| 71 | ridgwayi | Netherland Antilles | Netherlands Antilles-St.Marteen | museum/tp | 11/02/1905 | RMNH.AVES.162906 | KT123853 |  | Naturalis Biodiversity Centre - Leiden, NL (Becky Desjardins) |
| 72 | ridgwayi | Netherland Antilles | Netherlands Antilles-Bonaire | museum/tp | 25/11/1978 | ZMA.AVES.32804 | KT123850 |  | Naturalis Biodiversity Centre - Leiden, NL (Becky Desjardins) |
| 73 | ridgwayi | Netherland Antilles | Netherlands Antilles-Bonaire | museum/tp | 28/11/1951 | ZMA.AVES.11423 | KT123808 |  | Naturalis Biodiversity Centre - Leiden, NL (Becky Desjardins) |
| 74 | ridgwayi | Netherland Antilles | Netherlands Antilles-Bonaire | museum/tp | 01/06/1905 | ZMA.AVES.33694 | KT123809 |  | Naturalis Biodiversity Centre - Leiden, NL (Becky Desjardins) |
| 76 | ridgwayi | Bahamas | Great Inagua island | museum/tp | 10/03/1888 | BMNH 1906.12.7.624 | KT123858 |  | Natural History Museum - London (Mark Adams) |
| 77 | ridgwayi | Bahamas | unknown | museum/tp | 03/03/1902 | BMNH 1924.4.10.188 | KT123760 |  | Natural History Museum - London (Mark Adams) |
| 78 | ridgwayi | Bahamas | unknown | museum/tp | 06/12/1902 | BMNH 1924.4.10.189 | KT123761 |  | Natural History Museum - London (Mark Adams) |
| 79 | carolinensis | USA | Florida-Torch key | museum/tp | 01/01/1883 | RMNH.AVES.162900 | KT123762 |  | Naturalis Biodiversity Centre - Leiden, NL (Becky Desjardins) |
| 80 | carolinensis | USA | Florida-Spanish key | museum/tp | 01/01/1883 | RMNH.AVES.163190 | KT123763 |  | Naturalis Biodiversity Centre - Leiden, NL (Becky Desjardins) |
| 81 | carolinensis | USA | Florida-Spanish key | museum/tp | 01/01/1883 | RMNH.AVES.163331 | KT123764 |  | Naturalis Biodiversity Centre - Leiden, NL (Becky Desjardins) |
| 144 | carolinensis | USA | unknown | museum/tp | 16/04/1836 | RMNH.AVES.162896 | KT123795 |  | Naturalis Biodiversity Centre - Leiden, NL (Becky Desjardins) |
| 150 | carolinensis | Suriname | Matapica | museum/tp | 19/08/1961 | RMNH.33233 | KT123848 |  | Naturalis Biodiversity Centre - Leiden, NL (Becky Desjardins) |
| 151 | carolinensis | Suriname | Matapica | museum/tp | 04/08/1962 | RMNH.33831 | KT123673 |  | Naturalis Biodiversity Centre - Leiden, NL (Becky Desjardins) |
| 154 | carolinensis | Caribbean | Netherlands Antilles-Aruba island (origin Virginia) | museum/tp | 01/02/1979 | 608-39789 | KT123680 |  | Naturalis Biodiversity Centre - Leiden, NL (Becky Desjardins) |
| 199 | carolinensis | USA | Oregon | fresh/wb | 01/07/1999 | A 2431 | KT123729 |  | University of Greifswald - collection of M. Martell (Martin Haase) |
| 200 | carolinensis | USA | Oregon | fresh/wb | 01/07/1999 | A 2435 | KT123730 |  | University of Greifswald - collection of M. Martell (Martin Haase) |
| 216 | carolinensis | USA | Massachussetts-Westport | fresh/db | 17/05/2012 | 0928-09931 | KT123805 | KT123896 | Alan Poole (Cornell Lab of Ornithology - Ithaca, NY) |
| 217 | carolinensis | USA | Massachussetts-Westport | fresh/db | 29/05/2012 | no code | KT123682 |  | Alan Poole (Cornell Lab of Ornithology - Ithaca, NY) |
| 218 | carolinensis | USA | Massachussetts-Westport | fresh/db | 29/05/2012 | 0928-09932 | KT123854 | KT123863 | Yula Kapetanankos & Alan Poole (Cornell Lab of Ornithology - Ithaca, NY) |
| 219 | carolinensis | USA | Massachussetts-Westport | fresh/db | 30/05/2012 | no code | KT123676 |  | Yula Kapetanankos & Alan Poole (Cornell Lab of Ornithology - Ithaca, NY) |
| 220 | carolinensis | USA | Massachussetts-Westport | fresh/db | 30/05/2012 | no code | KT123681 |  | Yula Kapetanankos & Alan Poole (Cornell Lab of Ornithology - Ithaca, NY) |
| 222 | carolinensis | USA | Massachussetts-Westport | fresh/db | 07/06/2012 | no code | KT123745 | KT123883 | Yula Kapetanankos & Alan Poole (Cornell Lab of Ornithology - Ithaca, NY) |
| 224 | carolinensis | USA | Massachussetts-Westport | fresh/db | NO DATE | no code | KT123806 |  | Alan Poole (Cornell Lab of Ornithology - Ithaca, NY) |
| b | carolinensis | USA | unknown | sequence/fs | NO DATE | no code | AY987232 |  | Lerner & Mindell (2005) |
| C | carolinensis | USA | unknown | sequence/fs | NO DATE | no code | EU167008 |  | Lerner & Mindell (2005) |
| 165 | haliaetus | Russia | Magadan region | museum/tp | 20/08/1936 | R-31411 | KT123751 |  | Zoological Museum of Moscow, Russia (Mikhail Kalyakin) |
| 166 | haliaetus | Russia | Baikal lake | museum/tp | 22/05/1959 | R-101447 | KT123752 |  | Zoological Museum of Moscow, Russia (Mikhail Kalyakin) |
| 167 | haliaetus | Russia | Kuriles islands | museum/tp | 01/01/1948 | R-79411 | KT123849 |  | Zoological Museum of Moscow, Russia (Mikhail Kalyakin) |
| 170 | haliaetus | Russia | Magadan region | museum/tp | 20/08/1936 | R-31410 | KT123857 |  | Zoological Museum of Moscow, Russia (Mikhail Kalyakin) |
| 171 | haliaetus | Russia | Khabarovsky region | museum/tp | 05/05/1965 | R-92511 | KT123674 | KT123894 | Zoological Museum of Moscow, Russia (Mikhail Kalyakin) |
| 173 | haliaetus | Russia | Primorskii region | museum/tp | 20/04/1962 | R-90372 | KT123755 |  | Zoological Museum of Moscow, Russia (Mikhail Kalyakin) |
| 174 | haliaetus | Mongolia | unknown | museum/tp | 29/06/1903 | R-4500 | KT123758 |  | Zoological Museum of Moscow, Russia (Mikhail Kalyakin) |
| 175 | haliaetus | Russia | Tuva region | museum/tp | 17/08/1902 | R-28728 | KT123756 |  | Zoological Museum of Moscow, Russia (Mikhail Kalyakin) |
| 176 | haliaetus | Russia | Khabarovsky region | museum/tp | 25/06/1910 | R-28723 | KT123757 |  | Zoological Museum of Moscow, Russia (Mikhail Kalyakin) |
| 178 | haliaetus | Japan | Ota-ku | fresh/ms | 22/10/2008 | 2008-265-NSMT-8057 | KT123708 | KT123893 | National Museum of Nature and Science, Amakubo - JP (Isao Nishiumi ) |
| 179 | haliaetus | Japan | Hokkaido | fresh/ms | 04/07/2008 | KUS14-NSMT-9259 | KT123709 | KT123895 | National Museum of Nature and Science, Amakubo - JP (Isao Nishiumi ) |
| 180 | haliaetus | Japan | Hokkaido | fresh/ms | 04/07/2008 | KUS15-NSMT-9260 | KT123710 | KT123859 | National Museum of Nature and Science, Amakubo - JP (Isao Nishiumi ) |
| 181 | haliaetus | Japan | Tokoname-shi | fresh/ms | 28/03/2011 | 2011.106-NSMT-50628 | KT123711 | KT123861 | National Museum of Nature and Science, Amakubo - JP (Isao Nishiumi ) |
| 182 | haliaetus | Japan | Ota-ku | fresh/ms | 06/12/2012 | 2013.8-NSMT-52035 | KT123712 | KT123888 | National Museum of Nature and Science, Amakubo - JP (Isao Nishiumi ) |
| 83 | cristatus | Indonesia | Sanghir island | museum/tp | 04/08/1865 | RMNH.AVES.163187 | KT123766 |  | Naturalis Biodiversity Centre - Leiden, NL (Becky Desjardins) |
| 84 | cristatus | Indonesia | Java (poeloe lantjang,batavia) | museum/tp | 11/08/1927 | RMNH.99462 | KT123767 |  | Naturalis Biodiversity Centre - Leiden, NL (Becky Desjardins) |
| 85 | cristatus | Indonesia | Java (poeloe lang,java sea) | museum/tp | 09/09/1906 | RMNH.99465 | KT123800 |  | Naturalis Biodiversity Centre - Leiden, NL (Becky Desjardins) |
| 86 | cristatus | Indonesia | Ceram sea (poeloe kasoeari) | museum/tp | 09/06/1910 | RMNH.AVES.163098 | KT123657 |  | Naturalis Biodiversity Centre - Leiden, NL (Becky Desjardins) |
| 87 | haliaetus | Indonesia | Sumatra (korintji,sandaron agang) | museum/tp | 14/07/1915 | RMNH.AVES.163143 | KT123798 |  | Naturalis Biodiversity Centre - Leiden, NL (Becky Desjardins) |
| 90 | cristatus | Indonesia | Morotai | museum/tp | 31/12/1861 | RMNH.AVES.162885 | KT123659 |  | Naturalis Biodiversity Centre - Leiden, NL (Becky Desjardins) |
| 91 | cristatus | Indonesia | Motie | museum/tp | 02/10/1863 | RMNH.AVES.162886 | KT123768 |  | Naturalis Biodiversity Centre - Leiden, NL (Becky Desjardins) |
| 93 | cristatus | Indonesia | Celebes (Buton) | museum/tp | 23/09/1948 | ZMA.AVES.47906 | KT123846 |  | Naturalis Biodiversity Centre - Leiden, NL (Becky Desjardins) |
| 94 | cristatus | Indonesia | Sanghir island | museum/tp | 24/01/1886 | RMNH.AVES.162903 | KT123660 |  | Naturalis Biodiversity Centre - Leiden, NL (Becky Desjardins) |
| 95 | cristatus | Indonesia | Siau island | museum/tp | NO DATE | RMNH.AVES.163100 | KT123769 |  | Naturalis Biodiversity Centre - Leiden, NL (Becky Desjardins) |
| 96 | cristatus | Indonesia | Tanahwangko, Minahasa, Celebes | museum/tp | 11/01/1940 | ZMA.AVES.47510 | KT123770 |  | Naturalis Biodiversity Centre - Leiden, NL (Becky Desjardins) |
| 97 | cristatus | Indonesia | Ceram, Kaibobo | museum/tp | NO DATE | RMNH.AVES.163238 | KT123661 |  | Naturalis Biodiversity Centre - Leiden, NL (Becky Desjardins) |
| 98 | cristatus | Indonesia | Borneo (Pagattan) | museum/tp | 02/09/1844 | RMNH.AVES.162908 | KT123771 |  | Naturalis Biodiversity Centre - Leiden, NL (Becky Desjardins) |
| 99 | cristatus | Indonesia | Ternate island (Molucca islands) | museum/tp | 24/04/1861 | RMNH.AVES.162938 | KT123772 |  | Naturalis Biodiversity Centre - Leiden, NL (Becky Desjardins) |
| 100 | cristatus | Indonesia | Ternate island (Molucca islands) | museum/tp | 30/04/1861 | RMNH.AVES.163251 | KT123796 |  | Naturalis Biodiversity Centre - Leiden, NL (Becky Desjardins) |
| 101 | cristatus | Indonesia | Celebes,Mara | museum/tp | 20/04/1914 | RMNH.AVES.162869 | KT123797 |  | Naturalis Biodiversity Centre - Leiden, NL (Becky Desjardins) |
| 102 | haliaetus | Indonesia | Pulau Batjan | museum/tp | 11/01/1861 | RMNH.AVES.163099 | KT123662 |  | Naturalis Biodiversity Centre - Leiden, NL (Becky Desjardins) |
| 103 | haliaetus | Indonesia | Java,Djampang Koelan | museum/tp | 17/02/1929 | RMNH.AVES.99451 | KT123677 |  | Naturalis Biodiversity Centre - Leiden, NL (Becky Desjardins) |
| 105 | cristatus | Indonesia | Java,Bokor,Batavia | museum/tp | 15/04/1912 | RMNH.AVES.99467 | KT123773 |  | Naturalis Biodiversity Centre - Leiden, NL (Becky Desjardins) |
| 106 | cristatus | Indonesia | Java,Bokor,Batavia | museum/tp | 15/04/1912 | RMNH.AVES.99457 | KT123663 |  | Naturalis Biodiversity Centre - Leiden, NL (Becky Desjardins) |
| 107 | cristatus | Indonesia | Java,Moeara angke | museum/tp | 27/12/1912 | RMNH.AVES.99458 | KT123774 |  | Naturalis Biodiversity Centre - Leiden, NL (Becky Desjardins) |
| 108 | cristatus | Indonesia | Java (poeloe lantjang,batavia) | museum/tp | 22/05/1922 | RMNH.AVES.99468 | KT123775 |  | Naturalis Biodiversity Centre - Leiden, NL (Becky Desjardins) |
| 109 | cristatus | Indonesia | Java | museum/tp | NO DATE | RMNH.AVES.99463 |  |  | Naturalis Biodiversity Centre - Leiden, NL (Becky Desjardins) |
| 110 | cristatus | Indonesia | Java,Bokor,Batavia | museum/tp | 06/10/1920 | RMNH.AVES.99453 | KT123664 |  | Naturalis Biodiversity Centre - Leiden, NL (Becky Desjardins) |
| 111 | cristatus | Indonesia | Aru islands | museum/tp | 19/06/1865 | RMNH.AVES.163322 | KT123776 |  | Naturalis Biodiversity Centre - Leiden, NL (Becky Desjardins) |
| 112 | cristatus | Indonesia | Babar island | museum/tp | 17/04/1898 | RMNH.AVES.163357 | KT123747 |  | Naturalis Biodiversity Centre - Leiden, NL (Becky Desjardins) |
| 113 | cristatus | Indonesia | Buru island (djikoe-merasa N.Beroe) | museum/tp | 25/07/1923 | RMNH.AVES.cat.29 | KT123665 |  | Naturalis Biodiversity Centre - Leiden, NL (Becky Desjardins) |
| 140 | cristatus | New Caledonia | unknown | museum/tp | NO DATE | RMNH.AVES.162873 | KT123792 |  | Naturalis Biodiversity Centre - Leiden, NL (Becky Desjardins) |
| 141 | cristatus | New Guinea | Sentani | museum/tp | 25/04/1903 | RMNH.AVES.163102 | KT123793 |  | Naturalis Biodiversity Centre - Leiden, NL (Becky Desjardins) |
| 142 | haliaetus | New Guinea | Dorek | museum/tp | NO DATE | RMNH.AVES.162884 | KT123671 |  | Naturalis Biodiversity Centre - Leiden, NL (Becky Desjardins) |
| 201 | cristatus | Australia | New South Wales | fresh/wb | 1998 | A 2447 | KT123731 | KT123862 | University of Greifswald - collection of M. Martell (Martin Haase) |
| 202 | cristatus | Australia | New South Wales | fresh/wb | 1998 | A 2448 | KT123732 | KT123890 | University of Greifswald - collection of M. Martell (Martin Haase) |
| 203 | cristatus | Australia | New South Wales | fresh/wb | 01/08/1998 | A 2450 | KT123733 | KT123892 | University of Greifswald - collection of M. Martell (Martin Haase) |
| 204 | cristatus | Australia | New South Wales | fresh/wb | 01/08/1998 | A 2451 | KT123734 | KT123886 | University of Greifswald - collection of M. Martell (Martin Haase) |
| 205 | cristatus | Australia | New South Wales | fresh/wb | 01/09/1998 | A 2452 | KT123804 | KT123889 | University of Greifswald - collection of M. Martell (Martin Haase) |
| 206 | cristatus | Australia | New South Wales | fresh/wb | 01/09/1998 | A 2453 | KT123735 | KT123885 | University of Greifswald - collection of M. Martell (Martin Haase) |
| 207 | cristatus | Australia | New South Wales | fresh/wb | 01/09/1998 | A 2454 | KT123736 | KT123891 | University of Greifswald - collection of M. Martell (Martin Haase) |
| 208 | cristatus | Australia | New South Wales | fresh/wb | 01/09/1998 | A 2455 | KT123737 | KT123887 | University of Greifswald - collection of M. Martell (Martin Haase) |
| 209 | cristatus | Australia | New South Wales | fresh/wb | 01/09/1998 | A 2456 | KT123738 | KT123884 | University of Greifswald - collection of M. Martell (Martin Haase) |
| a | cristatus | Australia | unknown | sequence/fs | NO DATE | no code | DQ780884 | DQ780884 | Lerner & Mindell (2005) |
| 1 | haliaetus | France | Loiret | fresh/ft | 2003 | BA10505 | KT123683 | KT123864 | Rolf Wahl (MNHN) |
| 2 | haliaetus | France | Loiret | fresh/ft | 2006 | BS14320 | KT123810 |  | Rolf Wahl (MNHN) |
| 3 | haliaetus | Latvia | Basi | fresh/ft | 08/07/2012 | ET3579 | KT123811 |  | Aigars Kalvans (LFN) |
| 4 | haliaetus | Latvia | Vaidavas lake | fresh/ft | 03/07/2012 | ET3505 | KT123812 | KT123874 | Aigars Kalvans (LFN) |
| 5 | haliaetus | France | Corsica-Cape Corse | fresh/db | 13/06/2012 | BS15569 (CAB) | KT123813 |  | Jean-Marie Dominici (PNRC) |
| 6 | haliaetus | France | Corsica-Scandola Reserve | fresh/db | 24/03/2012 | BS15576 (A02) | KT123814 |  | Flavio Monti & Jean-Marie Dominici (CEFE-PNRC) |
| 7 | haliaetus | Spain | Canary Islands-Tenerife Island | fresh/db | 16/06/2012 | (SN) | KT123815 | KT123882 | Manuel Siverio (GOHNIC) |
| 8 | haliaetus | Spain | Balearic Islands-Menorca Island | fresh/db | 19/06/2012 | (HU) | KT123816 |  | Rafel Triay (IME) |
| 9 | haliaetus | Morocco | Al-Hoceima National Park | fresh/db | 20/05/2012 | BS15552 (AAA) | KT123817 |  | Flavio Monti & Jean-Marie Dominici (CEFE-PNRC) |
| 10 | haliaetus | Morocco | Al-Hoceima National Park | fresh/db | 22/05/2012 | BS15554 (AAC) | KT123802 |  | Flavio Monti & Jean-Marie Dominici (CEFE-PNRC) |
| 11 | haliaetus | Morocco | Al-Hoceima National Park | fresh/db | 23/05/2012 | BS15556 (AAE) | KT123818 |  | Flavio Monti & Jean-Marie Dominici (CEFE-PNRC) |
| 12 | haliaetus | France | Corsica-Scandola Reserve | fresh/db | 29/05/2012 | BS15565 | KT123819 |  | Jean-Marie Dominici (PNRC) |
| 13 | haliaetus | France | Corsica | fresh/db | 29/05/2012 | BS15560 | KT123820 |  | Jean-Marie Dominici (PNRC) |
| 14 | haliaetus | France | Corsica | fresh/db | 29/05/2012 | BS15561 | KT123821 | KT123865 | Jean-Marie Dominici (PNRC) |
| 15 | haliaetus | France | Corsica-Porto gulf | fresh/db | 22/05/2012 | BS15573 (CAI) | KT123822 |  | Jean-Marie Dominici (PNRC) |
| 16 | haliaetus | France | Corsica-Galeria gulf | fresh/db | 01/05/2012 | BS15571 | KT123823 |  | Jean-Marie Dominici (PNRC) |
| 17 | haliaetus | France | Corsica-Scandola Reserve | fresh/db | 24/05/2012 | BS15563 | KT123824 |  | Jean-Marie Dominici (PNRC) |
| 18 | haliaetus | France | Corsica | fresh/db | 30/05/2012 | BS15566 | KT123825 |  | Jean-Marie Dominici (PNRC) |
| 19 | haliaetus | Latvia | Pukbu | fresh/ft | 02/07/2012 | ET3299 | KT123826 | KT123866 | Aigars Kalvans (LFN) |
| 20 | haliaetus | Latvia | Usmas lake | fresh/ft | 06/07/2012 | ET3546 | KT123827 |  | Aigars Kalvans (LFN) |
| 21 | haliaetus | Latvia | Usmas lake | fresh/ft | 06/07/2012 | ET3551 | KT123828 | KT123867 | Aigars Kalvans (LFN) |
| 22 | haliaetus | Latvia | Vecbebri | fresh/ft | 10/07/2012 | ET3593 | KT123829 | KT123868 | Aigars Kalvans (LFN) |
| 23 | haliaetus | Latvia | Baltmuiza | fresh/ft | 10/07/2012 | ET3600 | KT123830 |  | Aigars Kalvans (LFN) |
| 24 | haliaetus | Latvia | Apriki | fresh/ft | 08/07/2012 | ET3574 | KT123831 | KT123869 | Aigars Kalvans (LFN) |
| 25 | haliaetus | Latvia | Allarmuiza | fresh/ft | 09/07/2012 | ET3590 | KT123832 |  | Aigars Kalvans (LFN) |
| 26 | haliaetus | Latvia | Lubana lake | fresh/ft | 30/06/2012 | ET3269 | KT123833 | KT123870 | Aigars Kalvans (LFN) |
| 27 | haliaetus | France | Loiret | fresh/ft | 2006 | BS14321 | KT123834 |  | Rolf Wahl (MNHN) |
| 28 | haliaetus | France | Essonne | fresh/ft | 2006 | BS14332 | KT123835 |  | Rolf Wahl (MNHN) |
| 29 | haliaetus | France | Loir-et-Cher | fresh/ft | 2006 | BS14301 | KT123836 |  | Rolf Wahl (MNHN) |
| 30 | haliaetus | France | Loir-et-Cher | fresh/ft | 2006 | BS14309 | KT123837 |  | Rolf Wahl (MNHN) |
| 31 | haliaetus | France | Loiret | fresh/ft | 2006 | BS14326 | KT123838 |  | Rolf Wahl (MNHN) |
| 32 | haliaetus | France | Loir-et-Cher | fresh/ft | 2006 | BA10900 | KT123839 |  | Rolf Wahl (MNHN) |
| 33 | haliaetus | France | Loiret | fresh/ft | 2006 | BA10897 | KT123840 |  | Rolf Wahl (MNHN) |
| 34 | haliaetus | France | Loiret | fresh/ft | 2006 | BS14304 | KT123841 |  | Rolf Wahl (MNHN) |
| 35 | haliaetus | France | Loiret | fresh/ft | 2006 | BS14315 | KT123842 |  | Rolf Wahl (MNHN) |
| 36 | haliaetus | France | Loiret | fresh/ft | 2006 | BA10893 | KT123843 | KT123871 | Rolf Wahl (MNHN) |
| 37 | haliaetus | Spain | Canary Islands-La Gomera | fresh/ft | 01/06/2009 | S8 | KT123844 | KT123872 | Manuel Siverio (GOHNIC) |
| 38 | haliaetus | Spain | Canary Islands-Tenerife | fresh/ft | 23/05/2009 | S7 | KT123684 |  | Manuel Siverio (GOHNIC) |
| 39 | haliaetus | Italy | Maremma Regional Park | fresh/ft | 11/06/2012 | S7 | KT123845 | KT123873 | Flavio Monti & Andrea Sforzi (CEFE-MRP) |
| 41 | haliaetus | Cape Vert | Boavista | fresh/db | 01/04/2012 | 01 | KT123807 |  | Pedro López-Suárez (NCV) |
| 42 | haliaetus | Cape Vert | Boavista | fresh/db | 14/04/2012 | 02 | KT123803 |  | Pedro López-Suárez (NCV) |
| 43 | haliaetus | Spain | Canary Islands-Tenerife | fresh/db | 16/06/2012 | SP | KT123655 |  | Manuel Siverio (GOHNIC) |
| 44 | haliaetus | Morocco | Al-Hoceima National Park | fresh/db | JUNE 2012 | BS15558 (AAI) | KT123847 |  | Houssine Nibani (AGIR-PNAH) |
| 45 | haliaetus | Estonia | unknown | fresh/ft | 20/07/2012 | U4 | KT123685 | KT123875 | Urmas Sellis (EOS) |
| 46 | haliaetus | Estonia | unknown | fresh/ft | 19/07/2012 | S8 | KT123855 | KT123876 | Urmas Sellis (EOS) |
| 47 | haliaetus | Estonia | unknown | fresh/ft | 19/07/2012 | S9 | KT123686 | KT123877 | Urmas Sellis (EOS) |
| 48 | haliaetus | Estonia | unknown | fresh/ft | 19/07/2012 | S7 | KT123687 | KT123860 | Urmas Sellis (EOS) |
| 49 | haliaetus | Estonia | unknown | fresh/ft | 03/08/2012 | U5 | KT123688 | KT123878 | Urmas Sellis (EOS) |
| 50 | haliaetus | Estonia | unknown | fresh/ft | 03/08/2012 | U6 | KT123746 | KT123879 | Urmas Sellis (EOS) |
| 51 | haliaetus | Estonia | unknown | fresh/ft | 04/08/2012 | S0 | KT123851 | KT123880 | Urmas Sellis (EOS) |
| 52 | haliaetus | Estonia | unknown | fresh/ft | 19/07/2012 | U2 | KT123689 |  | Urmas Sellis (EOS) |
| 53 | haliaetus | Finland | Hame region | fresh/ft | 23/07/2012 | M-61572 | KT123690 | KT123881 | Pertti Saurola (FMNH) |
| 54 | haliaetus | Finland | Hame region | fresh/ft | 25/07/2012 | M-64274 | KT123691 |  | Pertti Saurola (FMNH) |
| 55 | haliaetus | Finland | Hame region | fresh/ft | 25/07/2012 | M-64268 | KT123656 |  | Pertti Saurola (FMNH) |
| 56 | haliaetus | Finland | Hame region | fresh/ft | 25/07/2012 | M-64270 | KT123692 |  | Pertti Saurola (FMNH) |
| 57 | haliaetus | Finland | Hame region | fresh/ft | 25/07/2012 | M-64272 | KT123693 |  | Pertti Saurola (FMNH) |
| 58 | haliaetus | Finland | Lapland | fresh/ft | 24/07/2012 | M-63753 | KT123694 |  | Harri Koskinen (FMNH) |
| 59 | haliaetus | Finland | Lapland | fresh/ft | 25/07/2012 | M-63756 | KT123695 |  | Harri Koskinen (FMNH) |
| 60 | haliaetus | Finland | Lapland | fresh/ft | 25/07/2012 | M-63760 | KT123696 |  | Harri Koskinen (FMNH) |
| 61 | haliaetus | Finland | Lapland | fresh/ft | 25/07/2012 | M-63763 | KT123697 |  | Harri Koskinen (FMNH) |
| 62 | haliaetus | Finland | Lapland | fresh/ft | 26/07/2012 | M-63765 | KT123698 |  | Harri Koskinen (FMNH) |
| 63 | haliaetus | Finland | Lapland | fresh/ft | 26/07/2012 | M-63827 | KT123699 |  | Harri Koskinen (FMNH) |
| 64 | haliaetus | Finland | Lapland | fresh/ft | 27/07/2012 | M-63830 | KT123700 |  | Harri Koskinen (FMNH) |
| 65 | haliaetus | Finland | Lapland | fresh/ft | 27/07/2012 | M-63832 | KT123701 |  | Harri Koskinen (FMNH) |
| 66 | haliaetus | Spain | Balearic Islands-Menorca Island | fresh/wb | 14/06/2000 | A | KT123702 |  | Rafel Triay (IME) |
| 67 | haliaetus | Spain | Balearic Islands-Menorca Island | fresh/wb | 18/06/2000 | R | KT123703 |  | Rafel Triay (IME) |
| 68 | haliaetus | Spain | Balearic Islands-Menorca Island | fresh/wb | 15/06/2000 | N | KT123704 |  | Rafel Triay (IME) |
| 69 | haliaetus | Spain | Balearic Islands-Menorca Island | fresh/wb | 07/06/2000 | Z | KT123705 |  | Rafel Triay (IME) |
| 82 | cristatus | India | Hindustan | museum/tp | NO DATE | RMNH.AVES.163189 | KT123765 |  | Naturalis Biodiversity Centre - Leiden, NL (Becky Desjardins) |
| 114 | haliaetus | Italy | Genova, airport (Italy) | museum/tp | 01/08/1990 | MSNG-54719 | KT123666 |  | Museo "Giacomo Doria" - Genova, Italy (Enrico Borgo) |
| 115 | haliaetus | Italy | Genova, Pegli (Italy) | museum/tp | 24/03/1957 | MSNG-36570 | KT123777 |  | Museo "Giacomo Doria" - Genova, Italy (Enrico Borgo) |
| 116 | haliaetus | Italy | Murialdo, Savona (Italy) | museum/tp | 28/09/1939 | MSNG-33574 | KT123778 |  | Museo "Giacomo Doria" - Genova, Italy (Enrico Borgo) |
| 117 | haliaetus | Italy | Genova, Caselli (Italy) | museum/tp | 30/03/1949 | MSNG-34783 | KT123667 |  | Museo "Giacomo Doria" - Genova, Italy (Enrico Borgo) |
| 118 | haliaetus | Italy | Montecastello Tanaro, Alessandria (Italy) | museum/tp | 01/09/1946 | MSNG-34459 | KT123668 |  | Museo "Giacomo Doria" - Genova, Italy (Enrico Borgo) |
| 119 | haliaetus | Italy | Genova, Sestri Levante (Italy) | museum/tp | 20/03/1962 | MSNG-38664 | KT123779 |  | Museo "Giacomo Doria" - Genova, Italy (Enrico Borgo) |
| 120 | haliaetus | Italy | Genova, Busalla torrente Scrivia (Italia) | museum/tp | 26/03/1951 | MSNG-35046 | KT123669 |  | Museo "Giacomo Doria" - Genova, Italy (Enrico Borgo) |
| 121 | haliaetus | Italy | Genova, Sestri Ponente (Italy) | museum/tp | 16/04/1879 | MSNG-33132 | KT123780 |  | Museo "Giacomo Doria" - Genova, Italy (Enrico Borgo) |
| 122 | haliaetus | Italy | Genova, Cornigliano (Italy) | museum/tp | 31/03/1960 | MSNG-52971 | KT123781 |  | Museo "Giacomo Doria" - Genova, Italy (Enrico Borgo) |
| 124 | haliaetus | Italy | Imola, Basso di Poggi (Italia) | museum/tp | NO DATE | MSNG-53333 | KT123782 |  | Museo "Giacomo Doria" - Genova, Italy (Enrico Borgo) |
| 125 | haliaetus | Italy | Genova, Borzoli (Italia) | museum/tp | 01/04/1875 | MSNG-24720 | KT123783 |  | Museo "Giacomo Doria" - Genova, Italy (Enrico Borgo) |
| 127 | haliaetus | Pakistan | Baluchistan | museum/tp | 22/01/1872 | BMNH 1874.11.23.9 | KT123679 |  | Natural History Museum - London (Mark Adams) |
| 128 | haliaetus | Yemen | Abdul-Kori | museum/tp | 04/12/2012 | BMNH 1899.8.11.113 | KT123784 |  | Natural History Museum - London (Mark Adams) |
| 129 | haliaetus | Iran | Tunb islands | museum/tp | 18/03/1921 | BMNH 1924.3.20.74 | KT123856 |  | Natural History Museum - London (Mark Adams) |
| 130 | haliaetus | Iraq | Mesopotamia,Tigris,Hamar lake | museum/tp | 20/05/1928 | BMNH 1933.2.16.304 | KT123748 |  | Natural History Museum - London (Mark Adams) |
| 131 | haliaetus | Saudi Arabia | Arabia,Jizan | museum/tp | 15/12/1936 | BMNH 1937.4.17.244 | KT123785 |  | Natural History Museum - London (Mark Adams) |
| 132 | haliaetus | Saudi Arabia | Arabia,Jedda | museum/tp | 15/06/1948 | BMNH 1948.56.3 | KT123670 |  | Natural History Museum - London (Mark Adams) |
| 133 | haliaetus | Saudi Arabia | Arabia,Jedda | museum/tp | 08/06/1947 | BMNH 1949.24.6 | KT123786 |  | Natural History Museum - London (Mark Adams) |
| 134 | haliaetus | Saudi Arabia | Saudi Arabia | museum/tp | 09/02/1927 | BMNH 1949.24.9 | KT123706 |  | Natural History Museum - London (Mark Adams) |
| 135 | haliaetus | Netherland | Texel island | museum/tp | 09/05/1907 | RMNH.AVES.163191 | KT123787 |  | Naturalis Biodiversity Centre - Leiden, NL (Becky Desjardins) |
| 136 | haliaetus | Netherland | Zandvoort | museum/tp | 06/05/1919 | RMNH.AVES.162901 | KT123788 |  | Naturalis Biodiversity Centre - Leiden, NL (Becky Desjardins) |
| 137 | haliaetus | Netherland | Hillegom | museum/tp | 03/06/1880 | RMNH.AVES.163279 | KT123789 |  | Naturalis Biodiversity Centre - Leiden, NL (Becky Desjardins) |
| 138 | haliaetus | Netherland | Noordwijk | museum/tp | 05/05/1862 | RMNH.AVES.163264 | KT123790 |  | Naturalis Biodiversity Centre - Leiden, NL (Becky Desjardins) |
| 139 | haliaetus | Netherland | Leiduin,Vogelenzong | museum/tp | 26/04/1878 | RMNH.AVES.163268 | KT123791 |  | Naturalis Biodiversity Centre - Leiden, NL (Becky Desjardins) |
| 143 | haliaetus | India | Nipaul | museum/tp | NO DATE | RMNH.AVES.163256 | KT123794 |  | Naturalis Biodiversity Centre - Leiden, NL (Becky Desjardins) |
| 149 | haliaetus | Italy | Imola, Pontedassio (Italy) (origin Spain) | museum/tp | 22/05/1995 | MSNG-54785 | KT123672 |  | Museo "Giacomo Doria" - Genova, Italy (Enrico Borgo) |
| 152 | haliaetus | Italy | Genova, Lerca (Italy) (origin Sweden) | museum/tp | 17/04/1958 | MSNG-54788 | KT123678 |  | Museo "Giacomo Doria" - Genova, Italy (Enrico Borgo) |
| 155 | haliaetus | Germany | Germany | museum/es | NO DATE | PH1 (red) | KT123801 |  | Ursula Hofle Hansen (CIA) |
| 156 | haliaetus | Germany | Germany | museum/es | NO DATE | PH2 (white) | KT123749 |  | Ursula Hofle Hansen (CIA) |
| 162 | haliaetus | Portugal | Portugal | museum/es | 1995 | 219-95-PH1 (red) | KT123750 |  | Ursula Hofle Hansen (CIA) |
| 163 | haliaetus | Portugal | Portugal | museum/es | 1995 | PH2 (red) | KT123759 |  | Ursula Hofle Hansen (CIA) |
| 164 | haliaetus | Portugal | Portugal | museum/es | 1995 | 000166-95-PH3 (white) | KT123799 |  | Ursula Hofle Hansen (CIA) |
| 168 | haliaetus | Russia | Urals | museum/tp | 11/05/1939 | R-50450 | KT123753 |  | Zoological Museum of Moscow, Russia (Mikhail Kalyakin) |
| 169 | haliaetus | Russia | Urals | museum/tp | 10/06/1940 | R-50539 | KT123754 |  | Zoological Museum of Moscow, Russia (Mikhail Kalyakin) |
| 172 | haliaetus | Russia | Urals | museum/tp | 20/05/1941 | R-56829 | KT123675 |  | Zoological Museum of Moscow, Russia (Mikhail Kalyakin) |
| 177 | haliaetus | Portugal | Portugal (origin Sweden) | fresh/ft | 01/04/2012 | P17 | KT123707 |  | Luis Plama (CIBIO) |
| 183 | haliaetus | Russia | Russia, Darwin state natural biosphere reserve | fresh/wb | 14/07/2013 | 1 | KT123713 |  | Miroslav Babushkin (DSNBR) |
| 184 | haliaetus | Russia | Russia, Darwin state natural biosphere reserve | fresh/wb | 14/07/2013 | 2 | KT123714 |  | Miroslav Babushkin (DSNBR) |
| 185 | haliaetus | Russia | Russia, Darwin state natural biosphere reserve | fresh/wb | 14/07/2013 | 3 | KT123715 |  | Miroslav Babushkin (DSNBR) |
| 186 | haliaetus | Russia | Russia, Darwin state natural biosphere reserve | fresh/wb | 15/07/2013 | 4 | KT123716 |  | Miroslav Babushkin (DSNBR) |
| 187 | haliaetus | Russia | Russia, Darwin state natural biosphere reserve | fresh/wb | 15/07/2013 | 5 | KT123717 |  | Miroslav Babushkin (DSNBR) |
| 188 | haliaetus | Russia | Russia, Darwin state natural biosphere reserve | fresh/wb | 15/07/2013 | 6 | KT123718 |  | Miroslav Babushkin (DSNBR) |
| 189 | haliaetus | Russia | Russia, Darwin state natural biosphere reserve | fresh/wb | 15/07/2013 | 7 | KT123719 |  | Miroslav Babushkin (DSNBR) |
| 190 | haliaetus | Russia | Russia, Darwin state natural biosphere reserve | fresh/wb | 14/07/2013 | 8 | KT123720 |  | Miroslav Babushkin (DSNBR) |
| 191 | haliaetus | Russia | Russia, Darwin state natural biosphere reserve | fresh/wb | 15/07/2013 | 9 | KT123721 |  | Miroslav Babushkin (DSNBR) |
| 192 | haliaetus | Russia | Russia, National park "Russkiy Sever" | fresh/wb | 16/07/2013 | 10 | KT123722 |  | Miroslav Babushkin (DSNBR) |
| 193 | haliaetus | Russia | Russia, National park "Russkiy Sever" | fresh/wb | 16/07/2013 | 11 | KT123723 |  | Miroslav Babushkin (DSNBR) |
| 194 | haliaetus | Russia | Russia, National park "Russkiy Sever" | fresh/wb | 16/07/2013 | 12 | KT123724 |  | Miroslav Babushkin (DSNBR) |
| 195 | haliaetus | Russia | Russia, National park "Russkiy Sever" | fresh/wb | 16/07/2013 | 13 | KT123725 |  | Miroslav Babushkin (DSNBR) |
| 196 | haliaetus | United Arab Emirates | United Arab Emirates | fresh/wb | 01/04/1999 | A 2418 | KT123726 |  | University of Greifswald - collection of M. Martell (Martin Haase) |
| 197 | haliaetus | United Arab Emirates | United Arab Emirates | fresh/wb | 01/04/1999 | A 2419 | KT123727 |  | University of Greifswald - collection of M. Martell (Martin Haase) |
| 198 | haliaetus | United Arab Emirates | United Arab Emirates | fresh/wb | 01/04/1999 | A 2420 | KT123728 |  | University of Greifswald - collection of M. Martell (Martin Haase) |
| 210 | haliaetus | Portugal | Portugal | fresh/wb | NO DATE | A 2457 - ZMUG RA02H | KT123739 |  | University of Greifswald - collection of M. Martell (Martin Haase) |
| 211 | haliaetus | Portugal | Portugal | fresh/wb | NO DATE | A 2458 - ZMUG RA02H | KT123740 |  | University of Greifswald - collection of M. Martell (Martin Haase) |
| 212 | haliaetus | Portugal | Portugal | fresh/wb | NO DATE | A 2459 - ZMUG RA02H | KT123741 |  | University of Greifswald - collection of M. Martell (Martin Haase) |
| 213 | haliaetus | Portugal | Portugal | fresh/wb | NO DATE | A 2460 - ZGMU RA02H | KT123742 |  | University of Greifswald - collection of M. Martell (Martin Haase) |
| 214 | haliaetus | Portugal | Portugal | fresh/wb | NO DATE | A 2461 - ZGMU RA02H | KT123743 |  | University of Greifswald - collection of M. Martell (Martin Haase) |
| 215 | haliaetus | Portugal | Portugal | fresh/wb | NO DATE | A 2462 - ZGMU RA02H | KT123744 |  | University of Greifswald - collection of M. Martell (Martin Haase) |
| d | haliaetus | Germany | Germany | sequence/fs | NO DATE | no code | AJ604503 |  | Lerner & Mindell (2005) |
| e | haliaetus | Israel | Israel | sequence/fs | NO DATE | no code | EU345523 |  | Lerner & Mindell (2005) |
|  |  |  |  |  |  |  |  |  |  |
