# Supplementary material for: Being cosmopolitan: evolutionary history and phylogeography of a specialized raptor, the Osprey Pandion haliaetus
Source: BMC Evol Biol. 2015 Nov 17;15:255. doi: 10.1186/s12862-015-0535-6 (PMC4650845; doi:10.1186/s12862-015-0535-6)
Supplement: Additional file 2: — Sampling for analyses. Number of individuals (N) and sequence length (L in base pairs) used for each analysis (phylogeny and network) and for each gene or concatenation of genes. nd means not done. (DOC 29 kb) [file 12862_2015_535_MOESM2_ESM.doc]

**Additional file 2: Sampling for analyses**

Number of individuals (N) and sequence length (L in base pairs) used for each analysis (phylogeny and network) and for each gene or concatenation of genes. nd means not done.

| Analysis |  | Cyt *b* | | ND2 | | Cyt *b* + ND2 | |
| --- | --- | --- | --- | --- | --- | --- | --- |
|  |  | N | L | N | L | N | L |
| Phylogeny | sequences | 209 | 1103 | 39 | 1078 | 38 | 2037 |
|  | haplotypes | 19 | 661 | nd | nd | 14 | 2037 |
| Network | sequences | 146 | 661 | nd | nd | nd | nd |
