# Supplementary material for: Being cosmopolitan: evolutionary history and phylogeography of a specialized raptor, the Osprey Pandion haliaetus
Source: BMC Evol Biol. 2015 Nov 17;15:255. doi: 10.1186/s12862-015-0535-6 (PMC4650845; doi:10.1186/s12862-015-0535-6)
Supplement: Additional file 3: — DNA amplification. Cytochrome b and ND2 primer names and sequences for amplification and sequencing. (DOC 32 kb) [file 12862_2015_535_MOESM3_ESM.doc]

**Additional file 3: DNA amplification**

Cytochrome *b* and ND2 primer names and sequences for amplification and sequencing.

| **cyt *b* Primers** | **Sequence** | **ND2 Primers** | **Sequence** |
| --- | --- | --- | --- |
| PANH-F1 | 5’- ATGGCCCCCAATCCTCGAAAATCACACC - 3’ | F13 | 5’- CCCATACCCCGAAAATGATGG - 3’ |
| PANH-F2 | 5’- AGTCAATAACTCCCTAATCGACC - 3’ | PHND2-F1 | 5’- GATCATCAGGACAGTGAGACATCACCC - 3’ |
| PANH-F6 | 5’- ACACAACCCTAGCCT TCTCATCC - 3’ | F17 | 5’- ACTAACAGGTTTCCTACCTAAGTGGC - 3’ |
| PANH-F10 | 5’- CCCTCATAGCAACAGCCT TC - 3’ | PHND2-R1 | 5’- GGCCTTCGGTTTTGGTTATCC - 3’ |
| PANH-R1 | 5’- TCTACTGAGAAACCTCCTCAGGCTC - 3’ | PHND2-R2 | 5’- AGTTGGTAGAGTTGGGGGGTAGTGTG - 3’ |
| PANH-R3  PANH-R5 | 5’- AACAGGTTGGGTGAGAACATGGATAGGG - 3’  5’ - CTTTGGTTTACAAGACCAATGT - 3’ |  |  |

For fresh samples, amplifications were conducted using the primers PANH-F1 and PANH-R5 for the Cytochrome *b* gene (1040 bp) and F13 and PHND2-R1 for the ND2 gene (1100 bp).

For museum samples, amplifications were conducted in several overlapping fragments.

- For the cytochrome *b*: a maximum of 752 bp were amplified using different sets of primers: PANHF1 - PANHR1 (513 bp)

or PANHF2 - PANHR1 (472 bp)

or PANHF6 - PANHR1 (340 bp)

or PANHF10 - PANHR1 (146 bp)

and PANHF10 -PANHR3 (384 bp) that overlapped by 146 bp

- For ND2: 805 bp were amplified using the primers PHND2-F1 and PHND2-R2 (673 bp) and, F17 and PHND2R1 (270 bp) that overlapped by 138 bp.
