# Supplementary material for: Being cosmopolitan: evolutionary history and phylogeography of a specialized raptor, the Osprey Pandion haliaetus
Source: BMC Evol Biol. 2015 Nov 17;15:255. doi: 10.1186/s12862-015-0535-6 (PMC4650845; doi:10.1186/s12862-015-0535-6)
Supplement: Additional file 4: — Phylogenetic trees of cyt b , ND2 and cyt b + ND2. Bayesian phylogenetic trees of ospreys constructed from: a) the cytochrome b (209 sequences, 1103 nucleotides) showing the four supported clades as well as the geographic origin of the samples; b) the ND2 (39 sequences, 1078 nucleotides) and (c) the concatenated genes (cyt b + ND2; 38 sequences, 2037 nucleotides). In all the trees, four species belonging to the Accipitridae family were used as outgroups. Branch lengths are proportional to the number of substitutions per site and \\ means that branches leading to outgroups have been reduced. For supported clades, bayesian posterior probabilities and ML bootstrap are indicated at nodes, respectively. (DOC 182 kb) [file 12862_2015_535_MOESM4_ESM.doc]

**Additional file 4: Phylogenetic trees of cyt *b*, ND2 and cyt *b*+ND2**

Bayesian phylogenetic trees of ospreys constructed from: a) the cytochrome *b* (209 sequences, 1103 nucleotides) showing the four supported clades as well as the geographic origin of the samples; b) the ND2 (39 sequences, 1078 nucleotides) and (c) the concatenated genes (cyt *b*+ND2; 38 sequences, 2037 nucleotides). In all the trees, four species belonging to the Accipitridae family were used as outgroups. Branch lengths are proportional to the number of substitutions per site and \\ means that branches leading to outgroups have been reduced. For supported clades, bayesian posterior probabilities and ML bootstrap are indicated at nodes, respectively.

**
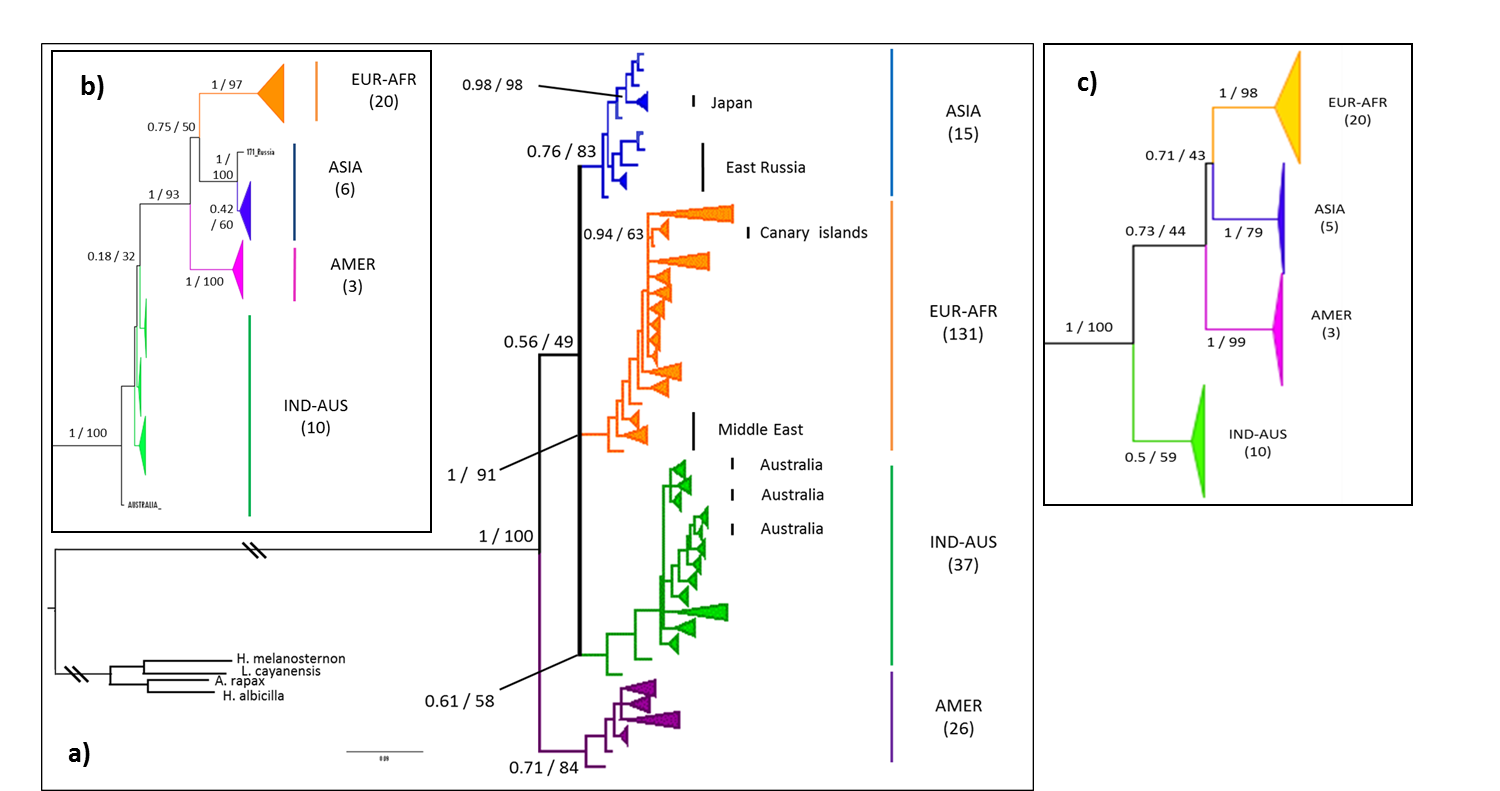
**
